# Bifunctional BODIPY-Clioquinol Copper Chelator with Multiple Anti-AD Properties

**DOI:** 10.3390/ijms262411876

**Published:** 2025-12-09

**Authors:** Daniil S. Abramchuk, Olga O. Krasnovskaya, Alevtina S. Voskresenskaya, Alexander N. Vaneev, Regina M. Kuanaeva, Vugara V. Mamed-Nabizade, Vasilii S. Kolmogorov, Olga I. Kechko, Vladimir A. Mitkevich, Alexander A. Makarov, Alexei A. Nastenko, Maxim A. Abakumov, Petr V. Gorelkin, Sergei V. Salikhov, Elena K. Beloglazkina, Alexander S. Erofeev

**Affiliations:** 1 Chemistry Department, Lomonosov Moscow State University, Leninskie Gory 1,3, 119991 Moscow, Russia; 2 Laboratory of Biophysics, National University of Science and Technology (MISIS), Leninskiy Prospect 4, 119049 Moscow, Russia; 3 Engelhardt Institute of Molecular Biology, Russian Academy of Sciences, Vavilov Str. 32, 117292 Moscow, Russia; 4 Department of Medical Nanobiotechnology, N.I. Pirogov Russian National Research Medical University, Ostrovityanova Str., 1, 6, 117997 Moscow, Russia

**Keywords:** boron dipyrromethene (BODIPY), clioquinol, Alzheimer’s disease (AD), amyloid beta (Aβ), optical visualization, copper chelator, scanning ion conductance microscopy (SICM)

## Abstract

Alzheimer’s disease (AD) is a worldwide problem due to the lack of effective therapy and accurate methods for timely diagnosis. The complexity of AD’s pathophysiology complicates the development of effective therapeutic agents, as most drugs act on only one therapeutic target, bypassing others. The design and development of multifunctional agents capable of altering metal ion-induced abnormalities, oxidative stress, and toxic beta amyloid (Aβ) aggregates is of interest. Herein, we report the first boron dipyrromethene (BODIPY) based bifunctional copper chelator with clioquinol, BDP-CLQ, capable of both optical detection of Aβ fibrils and copper chelation, with multiple anti-AD properties. Foremost, BDP-CLQ demonstrated a 3-fold and 5-fold fluorescence increase at 650 nm and 565 nm in the presence of Aβ and effective copper chelation (pK_d_ = 16.6 ± 0.3). In addition, BDP-CLQ demonstrated a potent inhibition of Aβ aggregation, reduction in Aβ-induced stiffness of neuronal cells, and antioxidant activity. BDP-CLQ is the first BODIPY-based fluorescent probe with multiple anti-AD activities, as well as the first clioquinol-based probe capable of Aβ optical visualization. This study demonstrates the prospects of the development of clioquinol-based theranostic probes since this allows combining several promising anti-AD actions in a single molecule and developing multi-targeted drugs.

## 1. Introduction

Alzheimer’s disease (AD) is the most common neurodegenerative disorder, characterized by memory loss and progressive cognitive deficiency [1]. The pathophysiology of AD is complicated and associated with different pathways, including deficiency in cholinergic transmission, defective metabolism of beta amyloid (Aβ), phosphorylation of tau proteins, deposition of neurofibrillary tangles (NFT), dyshomeostasis of metal ions, and the involvement of inflammatory and oxidative stress [2]. The development, design, and synthesis of anti-AD drugs capable of acting on multiple therapeutic targets is in high demand. The development of multifunctional agents capable of acting on several therapeutic targets at once—namely disaggregating Aβ, chelating metal cations, reducing oxidative stress, and reducing the neurotoxicity of Aβ plaques—is an extremely promising strategy [3,4,5].

The significant contribution of copper accumulation in brain tissue to the progression of AD was repeatedly discussed [6]. Abnormal copper level growth from 4.4 ± 1.5 μg/g in healthy brain tissue to 19 ± 6 μg/g in AD brain tissue is related to copper (II) ions binding with Asp1 and Ala2, as well as with Hys6 and Hys13/Hys14 residues of Aβ peptide, resulting in Aβ-Cu plaque formation [7]. The presence of abnormal copper (II) ions in the AD brain causes Aβ aggregation and oxidative stress, which in turn causes a Fenton-like reaction of copper cations with the release of reactive oxygen species (ROS) [8,9]. The application of copper chelators capable of copper level regulation in brain tissue is considered a promising approach for anti-AD drug design [10,11]. Thus, several copper chelators demonstrated the ability to reduce Aβ levels and lead to cognitive improvements in AD mice [12,13].

Clioquinol (5-chloro-7-iodo-8-hydroxyquinolinol, CLQ) is an FDA-approved drug for the treatment of skin infections with potent anti-AD therapeutic activity [14]. CLQ is a promising copper chelator capable of crossing the blood–brain barrier (BBB), and CLQ treatment has led to cognitive impairments in humans [15]. However, CLQ was rejected from phase II clinical trials due to its side effects [16,17]. In addition, CLQ moiety remains a promising scaffold for anti-AD drug development. For instance, several CLQ-based probes revealed an enhanced capability to reverse cognitive impairment in AD animal models [16,18].

The conjugation of Cu-chelating scaffolds with Aβ affine moiety, yielding bifunctional chelators (BFCs) capable of both Aβ binding and Cu ion chelating, is a promising therapeutic approach to AD treatment. Thus, several BFCs revealed the ability to reduce Aβ-induced oxidative stress and inhibit Aβ aggregation [19,20,21]. Additionally, the development of fluorophore-based BFCs capable of simultaneous diagnostic and therapeutic activity is of interest.

Optical probes for AD hallmark detection have been extensively developed due to the noninvasiveness, simplicity, and high sensitivity of this technique [22]. Boron dipyrromethene (BODIPY) fluorophores demonstrate the potent ability of AD hallmark fluorescent detection in vivo [23,24]. In addition, the development of BODIPY-based anti-AD theranostic probes with copper-chelating properties remains unexplored. To date, only one BODIPY-based probe with dipycolylamine (DPA) copper chelator was reported by Wang et al. [25]. Thus, we intend to take a step forward in this field and demonstrate the potential of BODIPY-based BFC investigation as a promising anti-AD theranostic probe.

Previously published bifunctional chelators typically contain Aβ-affine aromatic moieties of benzothiazole [26], stilbene [27], or azostilbene [28] and cyclic (NOTA, DOTA) [29,30] or non-cyclic (thiosemicarbazones) [21,31] metal-chelating scaffolds. In this study, we used the BODIPY fluorophore with a rotor unit as a copper-chelating scaffold, which is capable of enhancing fluorescence in the presence of Aβ due to the twisted intramolecular charge transfer (TICT) effect [32], and we also used CLQ, an anti-AD agent capable of disaggregating Aβ plaques, as a copper-chelating moiety. Interestingly, the combination of these two scaffolds in a single bifunctional chelator, BDP-CLQ, resulted in enhanced copper-chelating activity, strong disaggregating action, antioxidant activity, and the ability to reduce Aβ-induced neurotoxicity. This is the first example of a BODIPY-based bifunctional chelator with multiple anti-AD activities.

## 2. Results

### 2.1. Design of BDP-CLQ

**Figure 1:**
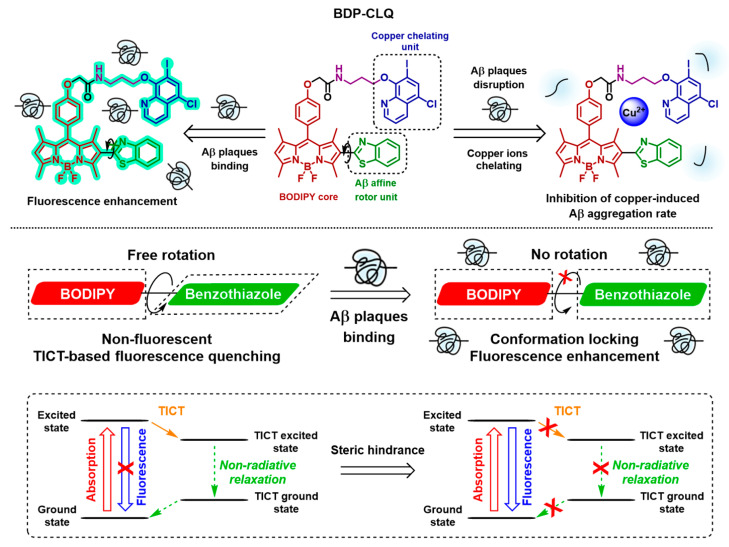
The twisted intramolecular charge transfer (TICT) mechanism of BDP-CLQ fluorescence enhancement upon binding with beta amyloid (Aβ) aggregates, and the design of bifunctional chelator (BFC) BDP-CLQ for optical visualization of Aβ aggregates and disruption of Aβ-Cu complexes.

The concept of fluorescent biomarkers implies a sharp fluorescence increase upon probe binding with a target. The fluorescence quenching of the diagnostic agent in an unbounded state is usually achieved by TICT. Fluorophore core coupling with a rotor unit enables non-radiative relaxation of the excited state of the probe by rotation of the rotor and stator relative to each other. In contrast, target binding leads to steric restrictions, TICT disturbance, and fluorescence enhancement (Figure 1) [33,34].

In the present study, we designed BDP-CLQ as a BODIPY fluorophore with a benzothiazole rotor unit for both optical detection of Aβ aggregates and copper-ion chelating. The interaction between the benzothiazole motif and the hydrophobic cavity of Aβ plaques can result in the conformational locking of the BDP-CLQ structure, leading to its fluorescence enhancement [32,35]. The copper-chelating CLQ scaffold was linked to the *meso*-position of the BODIPY core, enabling BDP-CLQ to affect the copper-induced AD-related processes (Figure 1).

**Scheme 1:**
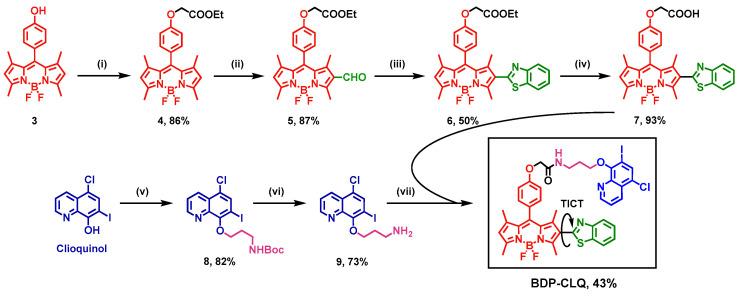
The synthesis of BDP-CLQ. (**i**) 2-bromoethylacetate, K_2_CO_3_, acetone, reflux, 2 h; (**ii**) POCl_3_, DMF, R.T., overnight; (**iii**) *o*-aminothiophenol, I_2_, DMF, 100 °C, 2 h; (**iv**) K_2_CO_3_, THF:H_2_O = 1:1, R.T., 24 h; (**v**) *tert*-butyl-3-bromopropyl carbamate, K_2_CO_3_, acetone, reflux, 6 h; (**vi**) TFA, DCM, R.T., 3 h; (**vii**) EDCl, N-hydroxysuccinimide, R.T., 24 h.

The bifunctional chelator BDP-CLQ was synthesized in 10 steps (Scheme 1 and Scheme S1). First, the BODIPY **3** was synthesized in 3 steps from acetoacetic ethyl ester and *p*-hydroxybenzaldehyde. Further modification of BODIPY **3** was conducted by consequent reactions of nucleophilic substitution and Vilsmeier–Haack formylation, resulting in BODIPYs **4** and **5**, respectively. Next, the condensation of BODIPY **5** with *o*-aminothiophenol yielded product **6**, which contains the benzothiazole group, while the hydrolysis of the ester resulted in product **7**. The BDP-CLQ was obtained by a carbodiimide synthesis with CLQ-propylamine **9**. In addition, BODIPY-based probe 5-MB-SZ [32] was synthesized in 3 steps as a reference compound for the determination of BDP-CLQ in vivo Aβ plaque detection efficacy (Scheme S2).

The structures of the substances were characterized by NMR ^1^H and ^13^C and high-resolution mass spectroscopy (Figures S1–S21 and S23–S25). The purity of BDP-CLQ > 99% was confirmed by HPLC analysis (Figure S22).

### 2.2. Photophysical Properties of BDP-CLQ

**Figure 2:**
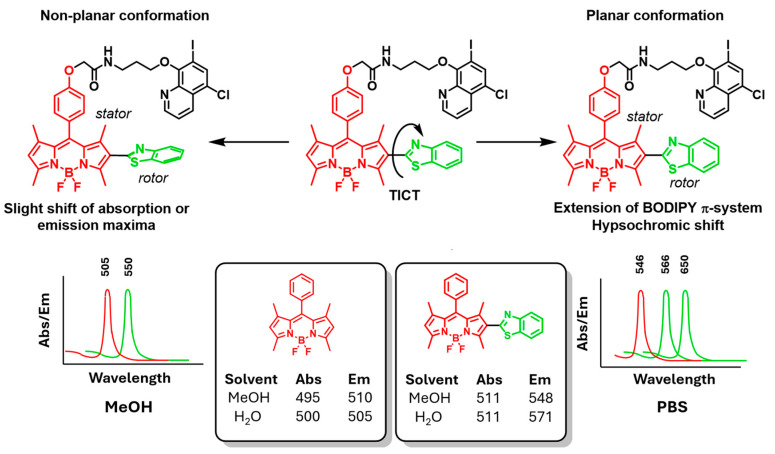
The influence of BDP-CLQ conformation on emission maxima shift [32].

**Figure 3:**
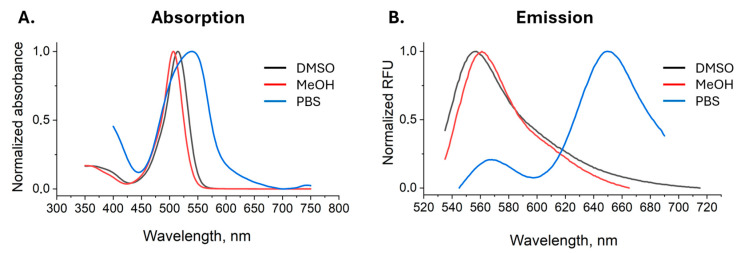
Normalized absorption (**A**) and emission (**B**) spectra of 100 µM BDP-CLQ in DMSO and MeOH, and 10 µM in PBS solutions.

BDP-CLQ is an optical probe with a TICT fluorescence quenching mechanism [34]. In particular, an intramolecular rotation of the rotor results in the stabilization of the planar conformation, a bathochromic shift in emission maximum, and a significant Stokes shift [36]. In non-polar solvents, BDP-CLQ showed similar absorption and emission maxima to those for BODIPY without a rotor unit, which is probably due to the perpendicular arrangement and the absence of π-conjugation between the BODIPY stator and benzothiazole rotor (Figure 2). In addition, in water, BDP-CLQ demonstrated a bathochromic shift in absorption, as well as two emission maxima at 565 and 650 nm, indicating the particular stabilization of the parallel arrangement of the BODIPY stator and benzothiazole rotor (Figure 2 and Figure 3 and Table 1). The participation of CLQ moiety in planar conformation stabilization could be suggested due to the absence of the second emission maximum for previously reported 2-benzothiazolyl BODIPY probes [32].

### 2.3. Copper-Chelating Properties of BDP-CLQ

As the copper-chelating properties of CLQ are the keystone of its therapeutic action, the investigation of the ability of BDP-CLQ to chelate Cu(II) ions under physiological conditions is of great interest. According to the previously reported data, the stoichiometry of the CLQ-Cu complex is CLQ_2_Cu, and the binding constant stability value is pK_d_ = 15.8 [37]. The stoichiometry of the BDP-CLQ-Cu complex was determined by UV-Vis BDP-CLQ titration with CuCl_2_ solution, and the ratio of BDP-CLQ and Cu^2+^ was obtained as 1:1 (Figure S26).

**Figure 4:**
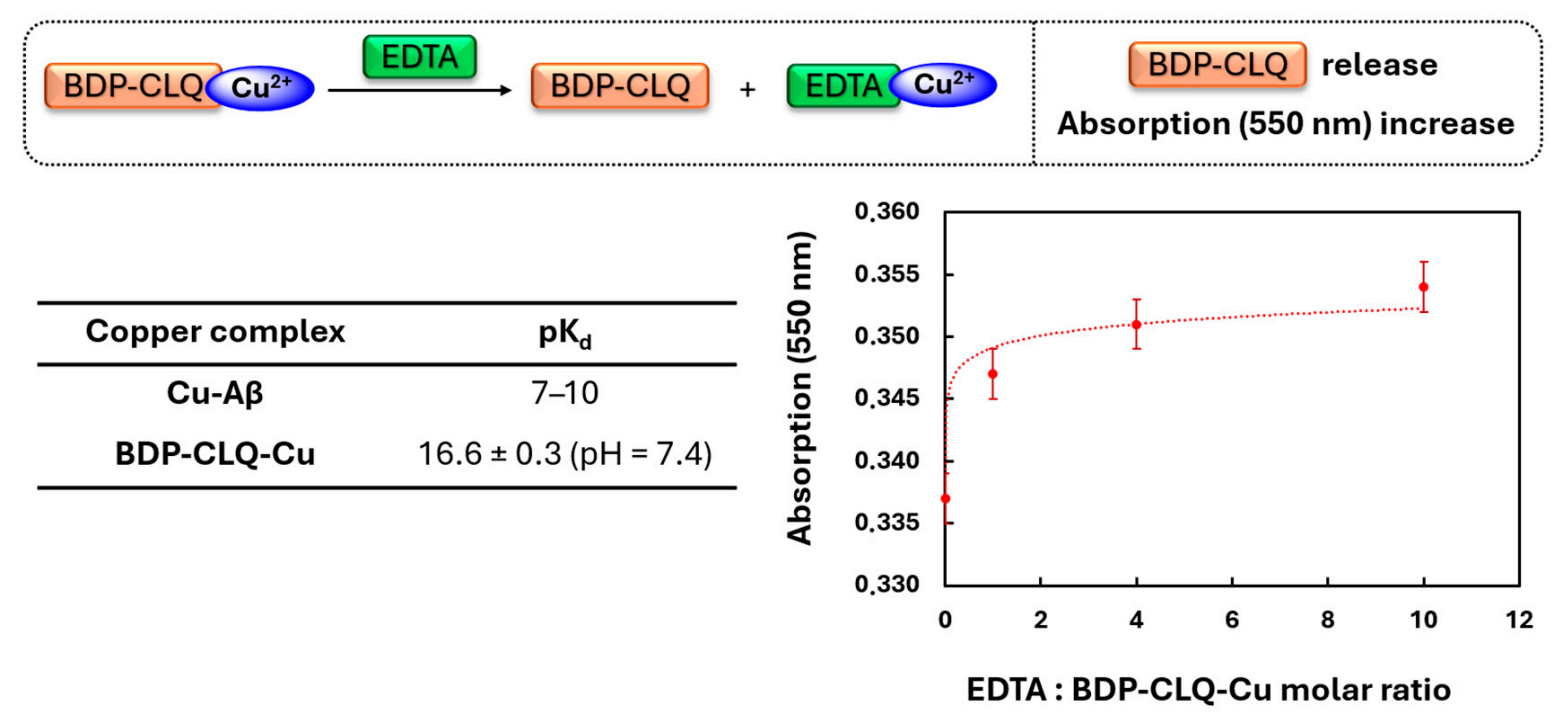
BDP-CLQ-Cu stability constant determination by competitive binding with EDTA.

Next, the stability constant of the BDP-CLQ-Cu complex was determined by competitive binding assay with EDTA (Figure 4) [27]. The pK_d_ value obtained was 16.6 ± 0.3 (PBS, 30% DMSO, pH = 7.4), which indicates higher BDP-CLQ copper-chelating activity in comparison to CLQ (pK_d_ = 15.8) [37]. BDP-CLQ copper-binding affinity is comparable with several reported NOTA-based BFCs [38]. In addition, this pK_d_ value indicates the ability of BDP-CLQ to disaggregate Cu-Aβ complexes, as its stability constant is pK_d_ = 7–10 depending on the conditions [39].

### 2.4. BDP-CLQ Cytotoxicity Measurement

To ensure the biocompatibility of BDP-CLQ, the cytotoxicity of BDP-CLQ was evaluated on human neuroblastoma cells both in the presence and absence of Cu(II) ions. The standard MTT assay demonstrated low toxicity of BDP-CLQ alone, as well as in the presence of Cu^2+^ on SH-SY5Y cells (IC_50_(BDP-CLQ) = 50.6 μM, IC_50_ (BDP-CLQ-Cu) > 50 μM), indicating the biosafety of BDP-CLQ (Figure S27).

### 2.5. Fluorescence Enhancement of BDP-CLQ upon Incubation with Beta Amyloid (Aβ_42_) Fibrils

**Figure 5:**
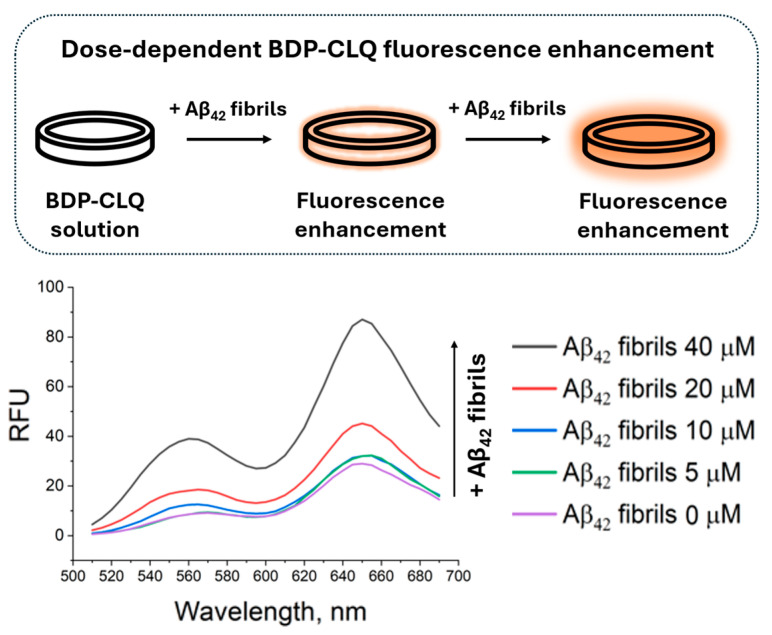
BDP-CLQ fluorescence enhancement upon incubation of 10 µM with Aβ_42_ fibrils.

The dose-dependent fluorescence enhancement upon probe binding with Aβ aggregates is essential for effective Aβ plaque optical detection [40]. To investigate the ability of BDP-CLQ to enhance its fluorescence in the presence of Aβ_42_ fibrils, the BDP-CLQ PBS solution was titrated with Aβ_42_ fibrils, and changes in fluorescence were registered. Thus, BDP-CLQ demonstrated a 5-fold, and 3-fold fluorescence intensity increase upon titration at 565 and 650 nm, respectively (Figure 5). In addition, a linear dependence between fluorescence intensity and Aβ_42_ concentration was observed (Figure S28). BDP-CLQ fluorescence increases upon Aβ binding, which correlates with the emission enhancement of other optical anti-AD theranostics [41,42], indicating the applicability of BDP-CLQ for Aβ plaque visualization. Furthermore, BDP-CLQ is the first CLQ-based anti-AD agent capable of fluorescent detection of Aβ fibrils.

### 2.6. Binding Affinity of BDP-CLQ Toward Aβ_42_ Aggregates

The affinity of BDP-CLQ for Aβ_42_ aggregates and monomers was measured by saturation binding assay and isothermal titration calorimetry (ITC). The saturation binding assay revealed a K_d_ value of BDP-CLQ for Aβ_42_ fibrils of ~1.45 ± 0.26 µM (Figure S29). In addition, the K_d_ value of BDP-CLQ for Aβ_42_ monomers in the ITC test and saturation binding assay was estimated to be 4.08 ± 0.20 µM and 2.18 ± 0.85 µM (Figures S29 and S30). It should be noted that the ITC technique cannot be applied for BDP-CLQ affinity measurement to Aβ fibrils, as Aβ aggregates represent a highly heterogeneous system. These results indicate a strong interaction between BDP-CLQ and Aβ species, as K_d_ values correlate with other anti-AD diagnostic and theranostic probes [43,44].

### 2.7. Inhibition of Aβ_42_ Aggregation with BDP-CLQ

As BFCs were repeatedly reported as effective inhibitors of Aβ aggregation, the ability of BDP-CLQ to prevent Aβ aggregation was investigated by atomic force microscopy (AFM) imaging [45,46,47]. The AFM imaging technique was used due to the unique opportunity to analyze the morphology, size, and quantity of Aβ plaques [48].

**Figure 6:**
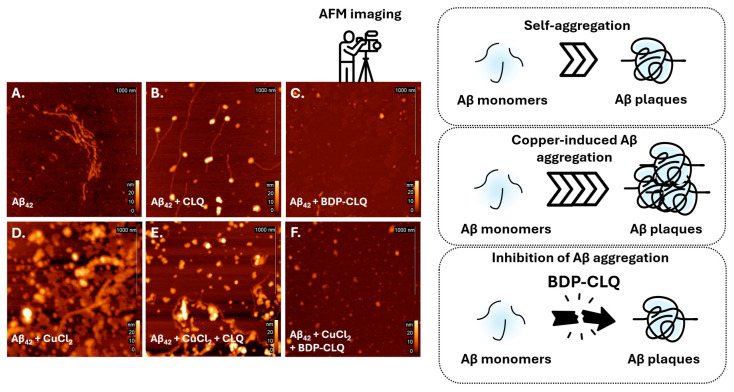
Atomic force microscopy (AFM) imaging of diverse Aβ_42_ assemblies. AFM images of self-aggregated Aβ_42_ fibrils (25 µM) (**A**), Aβ_42_ fibrils aggregated in the presence of clioquinol (CLQ) (25 µM) (**B**), Aβ_42_ fibrils with BDP-CLQ (50 µM) (**C**), Cu-induced Aβ_42_ fibrils (25 µM) (**D**), Aβ_42_ incubated with Cu^2+^ (25 µM) and CLQ (25 µM) (**E**), and Aβ_42_ incubated with Cu^2+^ (25 µM) and BDP-CLQ (50 µM) (**F**).

To evaluate the inhibitory activity of BDP-CLQ, monomeric Aβ_42_ was incubated both in the presence and absence of BDP-CLQ [49]. As CLQ was reported as a potent Aβ aggregation inhibitor, CLQ was used as the reference compound [50]. BDP-CLQ demonstrated the complete inhibition of Aβ aggregation both in the presence and absence of Cu(II) ions (Figure 6A,C,D,F and Figure S31A,C,D,F). In addition, CLQ also affected both copper-induced aggregation and self-aggregation of Aβ. However, the inhibitory activity of CLQ was lower in comparison with BDP-CLQ both in the presence and the absence of Cu(II) ions (Figure 6B,E and Figure S31B,E).

Thus, conjugation of the Aβ-disaggregating CLQ unit with BODIPY-benzothiazole scaffold led to the enhancement of Aβ disaggregation efficacy. This indicates the synergistic impact of BODIPY and CLQ on BDP-CLQ activity. In particular, the higher BDP-CLQ affinity for Cu(II) ion and Aβ aggregates probably leads to the higher BDP-CLQ inhibitory efficacy compared with CLQ.

### 2.8. The Investigation of BDP-CLQ Impact on Mechanical Properties of Aβ_42_-Affected Neuronal Cells

The progression of neurodegenerative diseases is also closely linked to cytoskeletal dysfunction. Aβ-induced neurotoxicity causes reorganization of the actin cytoskeleton—on one hand, through activation of kinases such as LIMK1, p38MAPK, CAMKII, and Rho/Cdc42 GTPases, and on the other hand, by increasing intracellular oxidative stress, which promotes glutathionylation and polymerization of actin filaments [51].

To investigate the ability of BDP-CLQ to decrease Aβ-induced neuronal cell membrane stiffness, a scanning ion conductance microscopy (SICM) technique was used. SICM imaging is a unique technique that allows simultaneous measurements of various important structural and functional parameters with nanometer resolution on living cells [52,53,54]. Previously we reported the first example of assessing the effect of a BFC with a copper-chelating unit on cell stiffness, administered using SICM [21].

**Figure 7:**
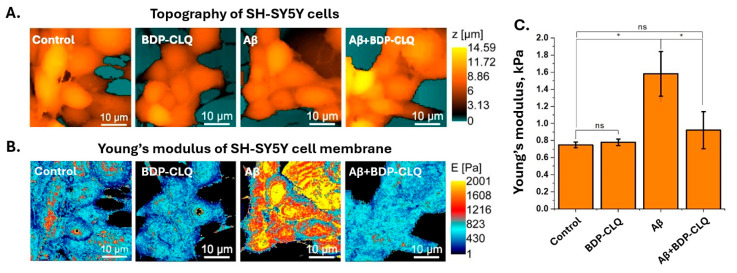
Topography (**A**) and Young’s modulus (**B**) maps of control SHSY-5Y cells, neuronal cells after incubation with 50 μM BDP-CLQ, Aβ_42_, and Aβ_42_ + 50 μM BDP-CLQ, and mean values of Young’s modulus of cell membranes in the samples (**C**). *—*p* < 0.05; ns—not significant (ANOVA test).

Expectedly, SH-SY5Y cells incubated with Aβ_42_ fibrils exhibited a significant increase in Young’s modulus up to 1.6 kPa, which correlates well with previous findings indicating that Aβ_42_ induces cytoskeletal stiffening [55]. The addition of BDP-CLQ to Aβ_42_-treated neuronal cells led to a decrease in cell stiffness, thereby leveling out the Aβ_42_-induced changes (Figure 7). These results demonstrate the effectiveness of BDP-CLQ as a neuroprotective agent. It should also be noted that SH-SY5Y cells incubated with BDP-CLQ alone demonstrated no significant deviation in stiffness from control cells (Figure 7).

Thus, BDP-CLQ is a potent modulator of Aβ_42_-induced mechanical dysfunction, restoring cell stiffness to near-physiological levels, which was confirmed by the SICM technique. The development of anti-AD agents with neuroprotective potential is a relevant task [56,57,58].

### 2.9. The Determination of BDP-CLQ Antioxidant Activity via Reduction in Intracellular Reactive Oxygen Species (ROS) Concentration

Generation of ROS and mitochondrial dysfunction are the main reasons for the multifaced toxicity observed in neuronal cells upon Aβ exposure [59]. Hence, the ability of BDP-CLQ to decrease intracellular ROS levels is essential for its antioxidant and neuroprotective activity [60,61]. To evaluate the ability of BDP-CLQ to reduce Aβ-induced oxidative stress in neuronal cells, we measured ROS levels directly in neuronal cells affected by Aβ, as well as in neuronal cells affected by Aβ and BDP-CLQ, via a Pt-coated carbon nanoelectrode capable of single-cell measurements [62,63,64].

**Figure 8:**
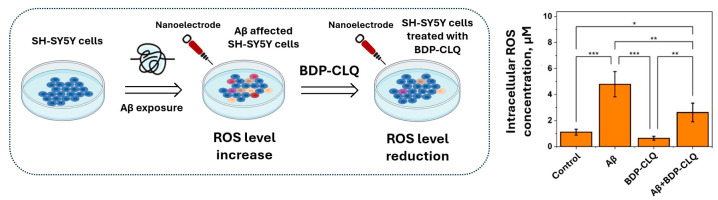
Electrochemical intracellular reactive oxygen species (ROS) level measurement in SH-SY5Y cells in the presence of Aβ species and BDP-CLQ. *—*p* < 0.05; **—*p* < 0.01; ***—*p* < 0.001 (ANOVA test).

The amperometric ROS level measurement of SH-SY5Y cells revealed an approximately 5-fold intracellular ROS-level increase upon incubation with Aβ_42_ aggregates, which confirms the extreme neurotoxicity of Aβ_42_ aggregates toward neuronal cells and the necessity for the use of antioxidant agents in anti-AD therapy. In addition, the incubation of SH-SY5Y cells with Aβ_42_ in the presence of BDP-CLQ resulted in only a 2.7-fold rise in ROS concentration (Figure 8). Thus, BDP-CLQ proved its high efficacy as an antioxidant agent against Aβ-induced oxidative stress.

Previously reported BFCs with antioxidant activity usually contain a phenol scaffold in their structure [27,28], and their antioxidant activity was demonstrated using DPPH (1,1-diphenyl-2-picryl-hydrazyl) or DCFH (dichlorofluorescein) [27,28]. In contrast, the antioxidant activity of BDP-CLQ was demonstrated directly on neuronal cells in the presence of Aβ species. It should also be noted that CLQ exhibits a moderate ability to reduce oxidative stress, while BDP-CLQ demonstrated promising antioxidative properties [65,66].

### 2.10. In Vivo Aβ Aggregates Detection by BDP-CLQ

Encouraged by the versatile BDP-CLQ activity in vitro, we evaluated the ability of BDP-CLQ to enhance its fluorescence in the brain tissue of APP/PS1 mice. The previously reported BODIPY-based probe 5-MB-SZ was used as a clioquinol-free reference compound, as it has been shown to efficiently accumulate in the brain [32]. A direct comparison of brain accumulation of the clioquinol-free BODIPY 5-MB-SZ and BDP-CLQ in APP/PS1 mice allows assessment of the influence of the clioquinol backbone on the ability of the BODIPY-based probe to cross the blood–brain barrier (BBB).

Thus, APP/PS1 mice (female, 10 months) and age-matched WT mice (male, 10 months) were administered a 22 μmol/kg (20 mg/kg) dose of BDP-CLQ by intraperitoneal injection; two control groups (APP/PS1, female, 10 months; and WT, male, 10 months) were administered a 22 μmol/kg (9 mg/kg) dose of 5-MB-SZ. The emission intensity measurement in APP/PS1 and WT mice was conducted within 90 min post-injection by IVIS technique.

As a result, both BDP-CLQ and 5-MB-SZ demonstrated enhanced fluorescence in APP/PS1 mice compared with WT mice (Figure S32), thus confirming that BDP-CLQ retains the tendency to accumulate in AD-affected brains, as previously shown for 5-MB-SZ. In addition, the following biodistribution assay demonstrated low BDP-CLQ BBB permeability (Figure S33). Thus, the conjugation of clioquinol with BODIPY generally reduces the ability of BDP-CLQ to overcome the BBB compared with the previously reported 5-MB-SZ; however, this issue might be solved by an intravenous injection through the retro-orbital sinus, which was previously proposed by Choi et al. for imaging Aβ aggregates and tau fibrils with heptamethine cyanine dye [67].

## 3. Discussion

Since AD pathology involves multiple molecular targets and pathways, the development of effective anti-AD drugs is a challenging task. Thus, the design of multi-targeted anti-AD drugs with potential for synergistic effects capable of affecting several AD-related mechanisms is relevant and an attractive challenge [68,69,70,71].

Thus, Xu et al. reported a potent multifunctional agent for the treatment of Alzheimer’s disease capable of inhibiting key enzymes and possessing antioxidant, antiaggregating, and copper-chelating properties [72,73]. Several tacrine derivatives were reported to act as multi-targeted drugs [74].

BFCs fundamentally possess metal chelation and Aβ interaction abilities [75]. However, several BFCs capable of metal chelation and antioxidant activity were also reported [27,28,76]. Thus, the development of BFCs with multiple anti-AD properties is of high relevance.

Herein, we report the design and synthesis of the first CLQ-based BFC BDP-CLQ capable of simultaneous dose-dependent fluorescence enhancement in the presence of Aβ, inhibition of Aβ aggregation, and neuroprotective activity against Aβ-induced toxicity and oxidative stress. Upon incubation with Aβ_42_ fibrils, BDP-CLQ demonstrated a 3-fold fluorescence intensity increase at 650 nm and a 5-fold emission enhancement at 565 nm (Figure 5). Thus, BDP-CLQ is the first CLQ-based theranostic agent capable of optical detection of Aβ plaques.

BDP-CLQ demonstrated a potent affinity for the copper (II) ion (pK_d_ = 16.6 ± 0.3), which is higher than that of the parent CLQ (pK_d_ is of 15.8) (Figure 4). The stability constant of the BDP-CLQ-Cu complex is comparable to the pK_d_ values of recently reported CLQ-based and ATSM-based BFCs capable of effective Cu(II) chelation under physiological conditions (pK_d_ in the range of 14 to 18) [77,78].

The affinity binding constants of BDP-CLQ toward Aβ_42_ fibrils and monomers appeared to be K_d_ = 1.45 ± 0.26 µM and K_d_ = 2.18 ± 0.85 µM, respectively (Figure S29). The affinity of BDP-CLQ toward Aβ species was estimated by fluorescent binding assay. These Aβ binding affinity values are in close accordance with those of previously reported optical anti-AD theranostics [44,79].

The ability of BDP-CLQ to inhibit both self- and copper-induced aggregation of Aβ was confirmed by AFM imaging (Figure 6 and Figure S31). Since the ability of CLQ to inhibit the Aβ aggregation was reported [50], a comparison of the anti-aggregating abilities of BDP-CLQ and CLQ was of interest. BDP-CLQ demonstrated the complete inhibition of both copper-induced and self-aggregation of Aβ_42_. In contrast, Aβ_42_ incubation with CLQ resulted in Aβ_42_ protofibrils formation, thus indicating that the anti-aggregation capacity of BDP-CLQ exceeds that of CLQ.

The accumulation of Aβ species in brain tissue induces significant stress on neurons through cell membrane interactions, and neuronal cells become rough and rigid [80]. BDP-CLQ demonstrated the capability to reduce Aβ-induced neuronal cell stiffness, which was demonstrated via SICM (Figure 7). Young’s modulus of SH-SY5Y cells increased approximately 2.1-fold upon incubation with Aβ, while in the presence of BDP-CLQ, the increase in neuronal cell stiffness was significantly lower (approximately 1.3-fold). These results indicate extremely promising BDP-CLQ neuroprotective activity against Aβ-induced neurotoxicity. Moreover, BDP-CLQ is the first optical BFC capable of protecting neuronal cell membranes against Aβ exposure.

The excellent antioxidant activity of BDP-CLQ was proven via a direct amperometric intracellular ROS level measurement in Aβ-affected SH-SY5Y cells. Thus, the addition of BDP-CLQ to Aβ-treated neuronal cells resulted in an approximately 2-fold decrease in ROS concentration (Figure 8). This study is the first direct amperometric determination of a BFC’s ability to reduce oxidative stress. Furthermore, BDP-CLQ is the first BODIPY-based anti-AD agent with antioxidant activity.

Thus, the modification of CLQ with BODIPY-benzothiazole scaffold resulted in synergistic anti-AD activity. BDP-CLQ demonstrated a higher affinity for the copper ion compared with CLQ. In addition, the inhibitory activity of BDP-CLQ toward Aβ aggregation was higher in comparison with CLQ both with and without the Cu(II) ion. Furthermore, BDP-CLQ demonstrated antioxidant activity in Aβ-affected neuronal cells and the ability to reduce SH-SY5Y cell membrane stiffness under Aβ exposure. In contrast, CLQ exhibited only a moderate ability to reduce Aβ-induced ROS generation [66].

To sum up, we report BODIPY-clioquinol BFC with multiple anti-AD activities. BDP-CLQ demonstrated extremely promising therapeutic and diagnostic activities in vitro. The diversified anti-Aβ activity of BDP-CLQ emphasizes the prospect of further development of CLQ-based anti-AD theranostic probes. Furthermore, the synergistic activity of BODIPY and CLQ moieties represents a promising approach for the development of optical theranostics with Cu chelators for AD treatment investigation.

## 4. Materials and Methods

### 4.1. Synthesis

Synthesis of ethyl 2-(4-(5,5-difluoro-1,3,7,9-tetramethyl-5H-4λ^4^,5λ^4^-dipyrrolo [1,2-c:2′,1′-f][1,3,2]diazaborinin-10-yl)phenoxy)acetate (**4**)

BODIPY **3** (0.4 g, 1.17 mmol) and anhydrous K_2_CO_3_ (0.365 g, 2.64 mmol) were suspended in 7 mL of dry acetone and stirred for 10 min at room temperature. Then ethyl 2-bromoacetate (1.30 mL, 1.173 mmol) was added. The resulting mixture was refluxed for 2 h. After cooling to room temperature, the mixture was diluted with DCM (50 mL), washed with water (2 × 30 mL), dried over Na_2_SO_4_, and evaporated to dryness under vacuum. Rufous red powder was obtained and purified by column chromatography with DCM as eluent, with an 86% yield.

The triplet at 1.30 ppm and quadruplet at 4.29 ppm correspond to the ethyl group of the ester. Singlets at 1.41 and 2.54 ppm represent pairs of methyl groups at the 1/7 and 3/5 positions of the BODIPY core. The singlet at 4.68 ppm with the intensity of 2H corresponds to the methylene group of acetic acid residue. The singlet at 5.97 ppm corresponds to the H atoms at the 2/6 positions of BODIPY fluorophore. Doublets at 7.02 and 7.18 ppm correspond to the *para*-substituted phenyl group at the *meso*-position of the BODIPY core.

^1^H NMR spectrum (400 MHz, CDCl_3_, δ, ppm): 1.30 (t, 3H); 1.41 (s, 6H); 2.54 (s, 6H); 4.29 (q, 2H); 4.68 (s, 2H); 5.97 (s, 2H); 7.02 (d, 2H); 7.18 (d, 2H).

^13^C NMR spectrum (100 MHz, CDCl_3_, δ, ppm): 14.14; 14.52; 29.68; 61.47; 65.41; 115.30; 121.17; 128.13; 129.31. 131.72; 141.35; 143.07; 155.38; 158.38; 168.48.

Synthesis of ethyl 2-(4-(5,5-difluoro-2-formyl-1,3,7,9-tetramethyl-5H-5λ^4^,6λ^4^-dipyrrolo [1,2- c:2′,1′-f][1,3,2]diazaborinin-10-yl)phenoxy)acetate (**5**)

DMF (5 mL) and POCl_3_ (3 mL) were charged into a flask under cooling in an ice-water bath and stirred under argon atmosphere for 1 h. BODIPY **4** (368 mg, 0.88 mmol) was dissolved in 5 mL of DCM and added to the mixture, left to stir overnight. The reaction mixture was slowly poured into a saturated aqueous solution of Na_2_CO_3_ (200 mL) cooled in an ice-water bath. When pH > 9 was reached, it was left under stirring for one hour, followed by extraction with DCM. The solution was dried over anhydrous Na_2_SO_4_, then the solvent was removed under reduced pressure. Scarlet-orange powder was obtained and purified by chromatography on a silica gel column with an eluting solvent of MeOH:DCM = 80:1 with an yield of 87%.

The triplet at 1.30 ppm and quadruplet at 4.28 ppm correspond to the ethyl group of the ester. Singlets with intensities of 3H at 1.45, 1.68, 2.59 and 2.80 ppm represent methyl groups at the 1, 3, 5 and 7 positions of the BODIPY core. The singlet at 4.70 ppm with the intensity of 2H corresponds to the methylene group of acetic acid residue. The singlet at 6.14 ppm corresponds to the only H atom at the 6 position of the BODIPY fluorophore. Doublets at 7.06 and 7.18 ppm correspond to the *para*-substituted phenyl group at the *meso*-position of the BODIPY core. The singlet at 9.99 ppm corresponds to the formyl group.

^1^H NMR spectrum (400 MHz, CDCl_3_, δ, ppm): 1.30 (t, 3H); 1.45 (s, 3H); 1.68 (s, 3H); 2.59 (s, 3H); 2.80 (s, 3H); 4.28 (m, 2H); 4.70 (s, 2H); 6.14 (s, 1H); 7.06 (d, 2H); 7.18 (d, 2H); 9.99 (s, 1H).

^13^C NMR spectrum (100 MHz, CDCl_3_, δ, ppm): 11.73; 12.97; 14.14; 15.03; 29.67; 61.53; 65.39; 115.63; 123.98; 127.19; 129.12; 142.76; 143.25; 147.21; 156.43; 158.79; 161.56; 168.33; 185.85.

LCMS(ESI): C_24_H_25_BF_2_N_2_O_4_ [M]^•+^, *m*/*z* calcd. 455.19, found 455.19.

Synthesis of ethyl 2-(4-(2-(benzo[d]thiazol-2-yl)-5,5-difluoro-1,3,7,9-tetramethyl-5H-5λ^4^,6λ^4^-dipyrrolo [1,2-c:2′,1′-f][1,3,2]diazaborinin-10-yl)phenoxy) acetate (**6**)

2-aminothiophenol (14 mg, 0.11 mmol) and BODIPY dye **5** (50 mg, 0.11 mmol) were mixed in DMF (3 mL); then, the iodine crystal (13 mg, 0.05 mmol) was added. The mixture was heated and stirred at 100 °C for 2 h. After that, the mixture was cooled to room temperature, followed by extraction with the solution of sodium thiosulfate (10%). The solution was dried over anhydrous Na_2_SO_4_; then the solvent was removed under reduced pressure. Red powder was obtained and purified by chromatography on a silica gel column with an eluting solvent of MeOH:DCM = 80:1, with a yield of 50%.

The triplet at 1.30 ppm and quadruplet at 4.29 ppm correspond to the ethyl group of the ester. Singlets with intensities of 3H at 1.45, 1.67, 2.60, and 2.84 ppm represent methyl groups at the 1, 3, 5, and 7 positions of the BODIPY core. The singlet at 4.70 ppm with the intensity of 2H corresponds to the methylene group of acetic acid residue. The singlet at 6.08 ppm corresponds to the only H atom at the 6 position of the BODIPY fluorophore. Doublets at 7.06 and 7.23 ppm correspond to the *para*-substituted phenyl group at the *meso*-position of the BODIPY core. Pairs of doublets (7.88 and 8.05 ppm) and triplets (7.38 and 7.48 ppm) correspond to the *ortho*-substituted phenyl ring of the benzothiazole unit.

^1^H NMR spectrum (400 MHz, CDCl_3_, δ, ppm): 1.30 (t, 3H); 1.45 (s, 3H); 1.67 (s, 3H); 2.60 (s, 3H); 2.84 (s, 3H); 4.29 (q, 2H); 4.70 (s, 2H); 6.08 (s, 1H); 7.06 (d, 2H); 7.23 (d, 2H); 7.38 (t, 1H); 7.48 (t, 1H); 7.88 (d, 1H); 8.05 (d, 1H).

^13^C NMR spectrum (100 MHz, CDCl_3_, δ, ppm): 13.18; 14.16; 14.89; 61.52; 65.40; 115.52; 121.21; 123.01; 124.82; 126.04; 127.80; 129.31; 135.34; 139.95; 142.49; 145.45; 153.36; 158.62; 161.08; 168.42.

LCMS(ESI) C_30_H_28_BF_2_N_3_O_3_S [M]^•+^, *m*/*z* calcd. 560.19, found 560.21.

Synthesis of 2-(4-(2-(benzo[d]thiazol-2-yl)-5,5-difluoro-1,3,7,9-tetramethyl-5H-5λ^4^,6λ^4^-dipyrrolo [1,2-c:2′,1′-f][1,3,2]diazaborinin-10-yl)phenoxy)acetic acid (**7**)

BODIPY **6** (30 mg, 0.055 mmol) and anhydrous K_2_CO_3_ (38 mg, 0.275 mmol) were suspended in a 6 mL solution of H_2_O:THF = 1:1. The mixture was stirred for 24 h at room temperature. Next, dilute hydrochloric acid (1M) was added to adjust pH to 2. The product was extracted with H_2_O and dried over anhydrous Na_2_SO_4_; then, the solvent was removed under reduced pressure. Deep violet powder was obtained with a 93% yield.

Singlets with intensities of 3H at 1.43, 1.65, 2.51 and 2.74 ppm represent methyl groups at the 1, 3, 5 and 7 positions of the BODIPY core. The singlet at 4.73 ppm with the intensity of 2H corresponds to the methylene group of acetic acid residue. The singlet at 6.34 ppm corresponds to the only H atom at the 6 position of the BODIPY fluorophore. Doublets at 7.12 and 7.34 ppm correspond to the H atoms of *para*-substituted phenyl ring at the *meso*-position of the BODIPY core. Pairs of doublets (8.01 and 8.10 ppm) and triplets (7.42 and 7.51 ppm) correspond to the *ortho*-substituted phenyl ring of the benzothiazole unit.

^1^H NMR spectrum (400 MHz, DMSO-d6, δ, ppm): 1.43 (s, 3H); 1.65 (s, 3H); 2.51 (s, 3H); 2.74 (s, 3H); 4.73 (s, 2H); 6.34 (s, 1H); 7.12 (d, 2H); 7.34 (d, 2H); 7.42 (t, 1H); 7.51 (t, 1H); 8.01 (d, 1H); 8.10 (d, 1H).

HRMS(ESI): C_28_H_24_BF_2_N_3_O_3_S [M]^•+^, *m*/*z* calcd. 532.1633 found 532.1676.

Synthesis of *tert*-butyl (3-((5-chloro-7-iodoquinolin-8-yl)oxy)propyl) carbamate (**8**)

To a solution of 5-chloro-7-iodoquinolin-8-ol (305 mg, 1 mmol) in acetone tert-butyl(3-bromopropyl)carbamate (286 mg, 1.2 mmol), anhydrous K_2_CO_3_ (414 mg, 3 mmol) were added, and then the mixture was heated to reflux for 6 h. The solution was cooled to room temperature and filtered, and the filtrate was dried under reduced pressure. Further purification was carried out by chromatography on a silica gel column with an eluting solvent of MeOH:DCM = 80:1. Dark-yellow oil was obtained with a yield of 82%.

The singlet with an intensity of 9H corresponds to the tert-butylcarbamate protecting group. Multiplets at 2.06, 3.60, and 4.30 ppm, each with 2H intensity, represent the sequence of methylene units. The broad singlet at 7.38 ppm corresponds to the H atom of the amide group. Two doublets and the doublet of doublets, each with 1H intensity at 7.57, 8.53, and 9.00 ppm, correspond to the pyridine ring of CLQ. The singlet at 7.94 corresponds to the H atom of the phenyl ring of CLQ moiety.

^1^H NMR spectrum (400 MHz, CDCl_3_, δ, ppm): 1.50 (s, 9H); 2.06 (t, 2H); 3.60 (q, 2H); 4.30 (t, 2H); 7.38 (br.s, 1H); 7.57 (dd, 1H); 7.94 (s, 1H); 8.53 (d, 1H); 9.00 (d, 1H).

Synthesis of 3-((5-chloro-7-iodoquinolin-8-yl)oxy)propan-1-amine (**9**)

*Tert*-butyl (3-((5-chloro-7-iodoquinolin-8-yl)oxy)propyl)carbamate **8** (300 mg, 0.648 mmol) was dissolved in DCM (6 mL). TFA (0.515 mL, 3.0 mmol) was added dropwise. The mixture was stirred for 3 h at room temperature. Then the reaction mixture was diluted with water and washed with a solution of NaOH (1 M). The product was dried over anhydrous Na_2_SO_4_, and then the solvent was removed under reduced pressure. The product was obtained as a dark goldenrod oil with a yield of 73%.

Multiplets at 2.10, 3.14 and 4.42 ppm, each with 2H intensity, represent the sequence of methylene units. Two doublets and the doublet of doublets, with 1H intensity each at 7.54, 8.51 and 8.95 ppm, correspond to the pyridine ring of CLQ. The singlet at 7.96 corresponds to the H atom of the phenyl ring of CLQ moiety.

^1^H NMR spectrum (400 MHz, CDCl_3_, δ, ppm): 2.10 (m, 2H); 3.14 (t, 2H); 4.42 (t, 2H); 7.54 (dd, 1H); 7.96 (s, 1H); 8.51 (d, 2H); 8.95 (d, 2H).

Synthesis of 2-(4-(2-(benzo[d]thiazol-2-yl)-5,5-difluoro-1,3,7,9-tetramethyl-5H-5λ^4^,6λ^4^-dipyrrolo [1,2-c:2′,1′-f][1,3,2]diazaborinin-10-yl)phenoxy)-N-(3-((5-chloro-7-iodoquinolin-8-yl)- oxy)propyl)acetamide (BDP-CLQ)

BODIPY **7** (42 mg, 0.0785 mmol) was dissolved in DCM (5 mL). EDCl (31 mg, 0.157 mmol) and NHS (18 mg, 0.157 mmol) were added to the reaction mixture. Acid activation was monitored by TLC. After 15 min of stirring, amine **9** (28.5 mg, 0.0785 mmol) was added. The resulting mixture was stirred overnight at room temperature. Then, the mixture was extracted with water (3 × 30 mL) and dried over anhydrous Na_2_SO_4_, and then the solvent was removed under reduced pressure. The crude product was purified by chromatography on a silica gel column with an eluting solvent of MeOH:DCM = 60:1. Product was obtained as a maroon-violet powder with a yield of 43%.

Singlets with intensities of 3H at 1.35, 1.44, 2.59, and 2.80 ppm represent methyl groups at the 1, 3, 5, and 7 positions of the BODIPY core. Multiplets at 2.10, 3.88, and 4.29 ppm, each with 2H intensity, represent the sequence of methylene units. The singlet at 4.63 ppm with the intensity of 2H corresponds to the methylene group of acetic acid residue. The singlet at 6.06 ppm corresponds to the only H atom at the 6 position of the BODIPY fluorophore. Doublets at 6.85 and 7.12 ppm correspond to the H atoms of the *para*-substituted phenyl ring at the *meso*-position of the BODIPY core. Triplets at 7.40 and 7.50 ppm and the doublet at 8.06 ppm correspond to the benzothiazol group. The multiplet at 7.90 corresponds to one H atom of the benzothiazol unit and the H atom of phenyl ring of CLQ moiety. Two doublets and the doublet of doublets, each with 1H intensity at 7.55, 8.54, and 9.00 ppm, correspond to the pyridine ring of CLQ. The triplet at 8.35 ppm corresponds to the H atom of the amide unit.

^1^H NMR spectrum (400 MHz, CDCl_3_, δ, ppm): 1.35 (s, 3H); 1.44 (s, 3H); 2.10 (m, 2H); 2.59 (s, 3H); 2.80 (s, 3H); 3.88 (q, 2H); 4.29 (t, 2H); 4.63 (s, 2H); 6.06 (s, 1H); 6.85 (d, 2H); 7.12 (d, 2H); 7.40 (t, 1H); 7.50 (t, 1H); 7.55 (dd, 1H); 7.90 (m, 2H); 8.06 (d, 1H); 8.35 (t, 1H); 8.54 (d, 1 H); 9.00 (d, 1 H).

^13^C NMR spectrum (100 MHz, CDCl_3_, δ, ppm): 12.60; 13.47; 14.46; 29.18; 35.90; 67.58; 71.89; 90.33; 115.03; 120.85; 121.94; 122.36; 122.68; 124.48; 125.67; 126.66; 127.19; 127.56; 129.02; 133.59; 134.68; 134.96; 141.70; 141.87; 150.38; 152.96; 154.34; 157.78; 160.58; 167.43.

HRMS (ESI): C_40_H_34_BClF_2_IN_5_O_3_S [M]^•+^, *m*/*z* calcd. 876.1210 found 876.1286.

Synthetic procedures and characterization of precursors are provided in the Supplementary Materials.

### 4.2. Absorption and Emission Spectra of BDP-CLQ

All absorbance and fluorescence measurements were performed using a Varioskan LUX plate reader (Thermo Scientific, Waltham, MA, USA). The BDP-CLQ DMSO solution (10 mM) was diluted with DMSO and MeOH to a final concentration of 100 μM, and with PBS to 10 μM. Then, 100 μL of each solution was placed on a black 96-well plate. The absorption spectra were recorded in a wavelength range of 350–750 nm. The fluorescence emission spectra of BDP-CLQ solutions were recorded in a wavelength range from 530 to 700 nm upon excitation at 515 nm for the DMSO solution, 510 nm for the MeOH solution, and 525 nm for the PBS solution.

### 4.3. Preparation of Aβ_42_ Samples

A preparation of a synthetic peptide of 42 amino acids β-amyloid Aβ_42_ (PepMic, Suzhou, China) was carried out using standard technology [81]. For this, 1 mg of the reagent was dissolved in 1,1,1,3,3,3-hexafluoro-2-propanol (Sigma, Saint Louis, MO, USA) on ice to reach a final peptide concentration of 1 mM in a glass vial. After dissolution, the solution was incubated for 1 h at room temperature to obtain peptide monomers. Then, the vial with the peptide was placed back on ice for 5 to 10 min, and the resulting solution was aliquoted into microtubes. Next, the tubes were left open to allow for evaporation of 1,1,1,3,3,3-hexafluoro-2-propanol. The remaining alcohol was evaporated using a rotary evaporator for 1 h, and the resulting films were stored at −70 °C.

A stock solution of Aβ_42_ peptide in DMSO (1.25 mM) was dissolved in filtered PBS 1× pH 7.4 to a final concentration of 100 μM. Aβ_42_ fibrils were prepared by incubation of Aβ_42_ monomer solution at 37 °C for 48 h.

### 4.4. Fluorescence Enhancement of BDP-CLQ Solution upon Incubation with Aβ_42_ Fibrils

The BDP-CLQ solution (10 μM in PBS 1×, pH = 7.4, 37 °C) was titrated with prepared Aβ_42_ fibril solution (0, 5, 10, 20, 40 μM). Emission spectra were measured in a wavelength range of 510–690 nm at λ_ex_ = 490 nm.

### 4.5. Stoichiometry of BDP-CLQ-Cu Complex Determination

UV-Vis titration was employed for the determination of the stoichiometry of the BDP-CLQ-Cu complex. The solution of BDP-CLQ (50 μM, 30% DMSO, pH 7.4) was titrated with small aliquots (20 μL) of CuCl_2_*2H_2_O (2.5 mM, 30% DMSO, pH 7.4) at room temperature. A total of 15 UV-Vis spectra were collected with a ratio of BDP-CLQ:Cu^2+^ from 10:1 to 1:2.

### 4.6. Stability Constant of BDP-CLQ-Cu Complex Determination

The stability constant of the BDP-CLQ-Cu complex was estimated by a competitive BFC binding assay with Na_2_H_2_EDTA [82]. A PBS solution of equimolar amounts of BDP-CLQ and CuCl_2_*H_2_O (50 μM, 30% DMSO, pH = 7.4) was incubated with EDTA (0.2, 0.4, 1.0, 2.0, and 4.0 eqv.) for 24 h at room temperature. Five UV−vis spectra were recorded with different ratios of BDP-CLQ-Cu and EDTA.

BDP-CLQ-Cu + EDTA = BDP-CLQ + EDTA-Cu

The stability constant for the BDP-CLQ-Cu complex was determined by fitting the observed absorbance data at 255 nm according to the following Equation [82]:

(1) $$y=\;\frac{K\times x}{C(1-x)}+x,$$

where y = [BDP-CLQ-Cu]/[EDTA]; x = saturation fraction = (A_L_ − A_O_)/(A_L_ − A_C_); A_L_, A_O_, and A_C_ are absorption values in the absence of EDTA, in the presence of EDTA, and at saturation with EDTA, respectively; C is the total concentration of BDP-CLQ; and *K* is the equilibrium constant. Thus, the stability constant can be estimated by the EDTA-Cu stability constant (K_d_ = 1.10 × 10^−16^ at pH = 7.4):

(2) $$K_{d}\;(\mathrm{BDP-CLQ-Cu})\;=\;K\;\times \;K_{d}\;(\mathrm{EDTA-Cu})$$

### 4.7. The Saturation Binding Affinity Assay of BDP-CLQ Toward Aβ_42_ Aggregates

A 5 μM solution of Aβ_42_ fibrils or monomers was titrated with BDP-CLQ solution (0, 0.05, 0.1, 0.25, 0.5, 1, 2, 5, 10, 15 μM) under near-physiological conditions (PBS, pH = 7.4), and emission spectra of samples were measured at λ_ex_(BDP-CLQ) = 525 nm, emission range 545–710 nm. The binding assay was performed with and without Aβ_42_ fibrils as the control. The experimental points were fitted by the saturation binding curve:

(3) $$y=\;\frac{P_{1}x}{P_{2}+x}$$

where y is a fluorescence of the sample (RFU), x is a concentration of BDP-CLQ (nM), $P_{1}$ is a fluorescence of BDP-CLQ solution upon saturation with Aβ_42_ fibrils, and $P_{2}$ is a binding affinity constant (K_d_).

### 4.8. The Determination of BDP-CLQ Binding Constant Toward Aβ_42_ Aggregates by Isothermal Titration Calorimetry

A 2.5 mM peptide stock solution was prepared by adding 100% anhydrous dimethyl sulfoxide (DMSO; Sigma-Aldrich, Saint Louis, MO, USA) to 0.25 mg of the peptide. The peptide was further diluted to the required concentration with a 50 mM Tris (pH 7.4). Only freshly prepared Aβ solution was used for all experiments. The binding constant and stoichiometry of BDP-CLQ binding to Aβ were measured using a MicroCal PEAQ-ITC instrument (Malvern Panalytical, Malvern, UK), as described previously [83]. Experiments were carried out at 25 °C in a 50 mM Tris (pH 7.4) containing 2% DMSO. Peptide concentration in the cell was 50 µM and ligand concentration in the syringe was 0.5 mM. A 40-μL syringe was used to inject 2.5 μL aliquots of BDP-CLQ into a 0.2 mL cell containing Aβ solution to achieve a complete binding isotherm. Heat of dilution was measured by injection of the ligand into the buffer solution and was subtracted from the binding isotherm. The resulting titration curve was fitted using MicroCal PEAQ-ITC Analysis Software v.1.41 (Malvern Panalytical, Malvern, UK).

### 4.9. The Effects of BDP-CLQ on Copper-Induced Aβ_42_ Aggregation

For the aggregation inhibition assay, 25 μM Aβ_42_ monomers were incubated with 25 μM CuCl_2_ solution for 2 min at room temperature, followed by incubation with 50 μM BDP-CLQ solution for 24 h at 37 °C.

Samples for AFM imaging were prepared according to the following procedure: 10 μL of each sample was added to a freshly cleaved mica sheet, incubated for 3 h, and then rinsed with ultrapure water and dried with argon. AFM measurements were performed in air using a NTEGRA II microscope (NT-MDT SI, Moscow, Russia) in semi-contact mode with silicon cantilevers (1.74 N/m, 90 kHz), scan size 2–10 μm, and 512 points. AFM images were processed with Femtoscan software v.2.4.26 (Advanced Technologies Center, Moscow, Russia) [84].

### 4.10. Cytotoxicity Assay

The SH-SY5Y cell line (ATCC, Manassas, VA, USA) was cultured in DMEM F/12 medium (PanEco, Moscow, Russia) containing 10% fetal bovine serum (FBS) (Gibco, Grand Island, NY, USA), 1× PenStrep (Gibco, USA), and 1× GlutaMax (Gibco, USA) and grown aseptically under standard conditions (37 °C, 5% CO_2_, humidified air atmosphere). Cell cultures were tested for the absence of mycoplasma. The cytotoxicity was measured using an MTT assay. The cells were seeded in wells of 96-well plates at 10,000 per well. After 24 h, the studied compound was added to the cells at an initial concentration of 200 μM for BDP-CLQ and 100 μM for BDP-CLQ + CuCl_2_, with serial dilutions threefold for the next 8 wells. As a control, DMSO at an initial concentration of 200 μM diluted 3 times was added to the wells. Cells were incubated with added compounds at 37 °C and 5% CO_2_ for 72 h (triplicate at each test compound concentration). After the incubation time, 100 μL of a pre-prepared MTT solution with a nutrient medium was added to each well at a ratio of 20 μL of MTT solution in DPBS (5 mg/mL) per 100 μL of medium. After 4 h of incubation, the medium containing MTT was removed, and 100 μL of DMSO was added to dissolve the reduced MTT-formazan adduct. The samples were incubated for 20 min on a shaker to completely dissolve the precipitated formazan in DMSO. Absorbance was measured using a Varioskan LUX multifunctional analyzer at 570 nm. The background was measured at 680 nm. The results were used to construct a four-parameter dose–response logistic curve and to estimate the IC_50_ value by Graphpad Prism software v.8.

### 4.11. Scanning Ion Conductance Microscopy (SICM)

SICM by ICAPPIC (ICAPPIC Ltd., London, UK) was used for topography mapping and estimation of Young’s modulus of SH-SY5Y cells. Nanopipettes with a typical tip radius of 45–50 nm were fabricated from borosilicate glass O.D. 1.0 mm, I.D. 0.5 mm (WPI, Hitchin, UK) using the laser puller P-2000 (Sutter Instruments, Novato, CA, USA). The nanopipette radius was calculated using the following theoretical model [85]:

(4) $$r=\frac{Io}{\pi Vktg(\alpha )},$$

where the half-cone angle *α* is 3 degrees, k is 1.35 S m^−1^, and V is the applied electrical potential of 200 mV.

The scanning procedure was performed in Hanks’ solution (Gibco, USA). Each experimental point had 20–30 cells scanned, with 3 independent replicates. For estimating Young’s modulus of living cells, a nanopipette was approached to the surface until the ion current through the tip was reduced by 2% from its initial value during scanning [86]. A noncontact topographic image was obtained at an ion current decrease of 0.5%, and a further two images were obtained at an ion current decrease (or setpoint) of 1% and 2%, corresponding to indentation depth produced by the intrinsic force at each setpoint. Then, the Young’s modulus was estimated by the following model:

(5) $$E=PA{\left(\frac{Ssub}{Scell}-1\right)}^{-1},$$

where *E* is the estimated Young’s modulus, *P* is the applied pressure, A is a constant depending on the nanopipette geometry, and Ssub and Scell are the slopes of the current–distance curve observed between the ion current decreases of 1% and 2% at the non-deformable surface (Ssub–substrate) and cell surface (Scell), respectively.

### 4.12. Amperometric Intracellular ROS Level Measurement in SH-SY5Y Cells

The total ROS concentration was determined by the amperometric method using Pt-nanoelectrodes. Commercially available, disk-shaped carbon nanoelectrodes isolated in quartz (ICAPPIC Limited, London, UK) with diameters of 60–100 nm were used to prepare Pt nanoelectrodes. Firstly, the carbon surface was etched in a 0.1 M NaOH, 10 mM KCl solution during 40 cycles of 10 s each (from 0 to +2200 mV) to create nanocavities. Further electrochemical deposition of platinum in the nanocavities was achieved by cycling from 0 to 800 mV, with a scan rate of 200 mV/s for 4 to 5 cycles in 2 mM H_2_PtCl_6_ solution in 0.1 M hydrochloric acid. Cyclic voltammetry from −800 to 800 mV with a scan rate of 400 mV/s in 1 mM ferrocenemethanol in PBS was used to control the electrode surface at all stages of fabrication. Prior to the measurements, each platinum nanoelectrode was calibrated using a series of standard H_2_O_2_ solutions (10^−7^, 10^−6^, 5 × 10^−6^, 10^−5^, 5 × 10^−5^, 10^−4^ M) at a potential of +800 mV vs. Ag/AgCl. Preparation of Pt-nanoelectrodes has been described in detail elsewhere [87,88].

SH-SY5Y (3 × 10^5^) cells were seeded in 35 mm Petri dishes and treated the next day with Aβ_42_/BDP-CLQ or Aβ_42_ with BDP-CLQ simultaneously. Aβ_42_ and BDP-CLQ were dissolved in DMSO. The final concentration of Aβ_42_/BDP-CLQ in the culture medium was 10 µM/50 µM (BDP-CLQ) with 4 h of incubation time. Untreated cells were used as a control, which was performed at the beginning and at the end of the experiment. Before the experiment, attached cells in Petri dishes were washed three times using Hanks’ Balanced Salt Solution to remove the growth media and traces of Aβ_42_/BDP-CLQ. A nanoelectrode penetrated the cells and measured the oxidation current of hydrogen peroxide. On average, about 15 cells were measured by 2–3 Pt electrodes in independent Petri dishes, with 3 independent replicates.

The setup for amperometric measurements included a PC connected to a system consisting of an ADC-DAC converter Axon Digidata 1550B (Axon Instruments, Union City, CA, USA) and patch-clamp amplifier MultiClamp 700B (Axon Instruments, USA). The working head of the amplifier was fixed on a PatchStar Micromanipulator (Scientifica, Uckfield, UK), which was placed near an inverted optical microscope, Nikon Eclipse TI-U (Nikon, Tokyo, Japan). A Pt nanoelectrode was fixed in a special holder on the working head of the amplifier. The potential difference between the Pt nanoelectrode and the reference (Ag/AgCl) electrode was recorded with the pClamp v.11 software suite (Molecular Devices, San Jose, CA, USA) and processed with Origin 2018 software.

### 4.13. In Vivo Aβ Plaques Visualization with BDP-CLQ by In Vivo Imaging System (IVIS)

In vivo imaging was carried out on IVIS Spectrum-CT (PerkinElmer, Shelton, CT, USA). APP/PS1 and WT were anesthetized with an isoflurane/air gas mixture (2%/98% isoflurane to air ratio). The previously reported 5-MB-SZ probe was used as a reference compound [32]. BDP-CLQ and 5-MB-SZ were injected i.p. at a dose of 22 μM/kg (10% DMSO, 90% propylenglycol), and imaging was performed in fluorescence mode with excitation filter = 535 nm and emission filter = 640 nm. Imaging was performed before injection and 90 min after the injection. ROI was chosen manually, and fluorescence intensity was measured using Living Image v.4.4 software (PerkinElmer, USA).

### 4.14. Biodistribution Assay

For the experiments, animals were anesthetized with an intraperitoneal injection of tiletamine (Zoletil, Virbac, Carros, France), followed by transcardial perfusion. For this, the right atrium was incised, a cannula was inserted into the left ventricle, and the bloodstream was flushed successively with sterile phosphate-buffered saline (Sigma-Aldrich, USA) and 10% HistoSafe buffered formalin (Biovitrum, Saint Petersburg, Russia). The organs were then removed, and their fluorescence was imaged ex vivo. ROI was chosen manually, and fluorescence intensity was measured using Living Image v.4.4 software (PerkinElmer, USA).

### 4.15. Statistical Analysis

All in vitro data were obtained in three independent experiments. Statistical analyses were performed using the ANOVA test. All plots show mean values ± SE. All tests assumed a normal distribution, and the statistical significance was set at *p* < 0.05.

## 5. Conclusions

In the present study, the novel BFC BDP-CLQ is reported to possess anti-Aβ neuroprotective properties and to be capable of Aβ optical detection, inhibition of Aβ aggregation, and antioxidant activity. BDP-CLQ is also the first CLQ-based BFC capable of optical detection of Aβ aggregates and the first BODIPY-based BFC with anti-AD theranostic activity. Moreover, BDP-CLQ is the first BFC capable of neuronal cell membrane anti-Aβ protective activity. Thus, we demonstrate the prospects of BODIPY-based anti-AD theranostic agents with copper-chelating properties, since the synergistic activity of BODIPY and CLQ moieties yields versatile anti-AD activity, which was not observed for either CLQ or BODIPY separately.

## Figures and Tables

**Table 1:** Photophysical properties of BDP-CLQ.

Solvent	Absorption, nm	Emission, nm	Stoke’s Shift, cm^−1^
MeOH	505	561	1976.7
DMSO	513	555	1475.2
PBS	540	565, 650	819.4; 3133.9

## Data Availability

The original contributions presented in this study are included in the article/Supplementary Material. Further inquiries can be directed to the corresponding author(s).

## Supplementary Materials

The following supporting information can be downloaded at https://www.mdpi.com/article/10.3390/ijms262411876/s1.

## Abbreviations

Aβ—beta-amyloid; AD—Alzheimer’s disease; AFM—atomic force microscopy; BBB-blood—brain barrier; BFC—bifunctional chelator; BODIPY—boron dipyrromethene; CLQ—clioquinol (5-chloro-7-iodo-8-hydroxyquinolinol); EDCl—1-Ethyl-3-(3-dimethyl aminopropyl) carbodiimide hydrochloride; FDA—Food and Drug Administration; ICT—intramolecular charge transfer; IVIS—in vivo imaging system; MTT—microtiter test; NFT—neurofibrillary tangles; NMR—nuclear magnetic resonance; PBS—phosphate-buffered solution; PET—positron emission tomography; ROS—reactive oxygen species; TICT—twisted intramolecular charge transfer.
