# Supplementary material for: Bifunctional BODIPY-Clioquinol Copper Chelator with Multiple Anti-AD Properties
Source: Int J Mol Sci. 2025 Dec 9;26(24):11876. doi: 10.3390/ijms262411876 (PMC12732972; doi:10.3390/ijms262411876)
Supplement: Supplementary file 1 [file ijms-26-11876-s001.zip › ijms-3934015-supplementary.pdf]

Supporting information

Bifunctional BODIPY-Clioquinol Copper Chelator with Multiple Anti-AD Properties

Daniil S. Abramchuk [1,2], Olga O. Krasnovskaya [1,2,*], Alevtina S. Voskresenskaya [1], Alexander N. Vaneev [1,2], Regina M. Kuanaeva [2], Vugara V. Mamed-Nabizade [2], Vasilii S. Kolmogorov [1,2], Olga I. Kechko [3], Vladimir A. Mitkevich [3], Alexander A. Makarov [3], Alexei A. Nastenko [2], Maxim A. Abakumov [4], Petr V. Gorelkin [2], Sergei V. Salikhov [2], Elena K. Beloglazkina [1] and Alexander S. Erofeev [1,2,*]

[1]Chemistry Department, Lomonosov Moscow State University, Leninskie gory 1,3, 119991, Moscow, Russia
[2]Laboratory of biophysics, National University of Science and Technology (MISIS), Leninskiy prospect 4, 119049, Moscow, Russia
[3]Engelhardt Institute of Molecular Biology, Vavilov str. 32, Russian Academy of Sciences, 119991 Moscow, Russia
[4] Department of Medical Nanobiotechnology, N.I. Pirogov Russian National Research Medical University, Ostrovityanova str., 1, 6, 117997, Moscow, Russia

Krasnovskayao@gmail.com
Erofeev@polly.phys.msu.ru

Table of Content

1. Synthetic procedures 4

2. NMR Spectra and HR-/LC-MS data 7

3. Copper chelating properties of BDP-CLQ 19

4. Cytotoxicity data 20

5. BDP-CLQ titration with Aβ_{42} fibrils solution 21

6. Binding affinity assay of BDP-CLQ to Aβ_{42} fibrils 21

7. Isothermal calorimetry titration assay 22

8. AFM imaging of BDP-CLQ inhibition of Aβ_{42} aggregation process 23

9. *In vivo* visualization of Aβ_{42} species 24

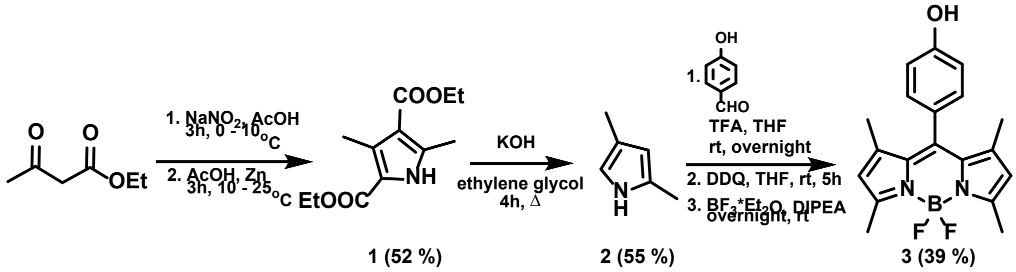

Scheme S1. Scheme of synthesis BODIPY **3**

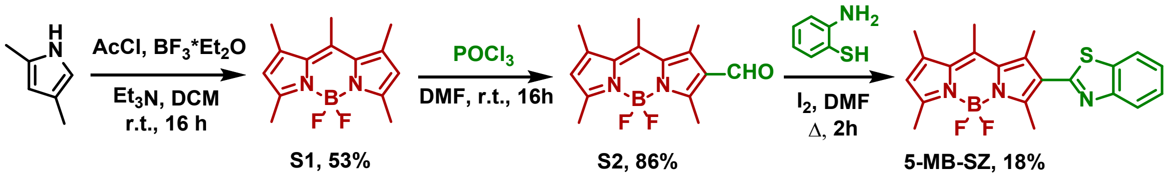

Scheme S2. Scheme of synthesis 5-MB-SZ

1. Synthetic procedures

Synthesis of diethyl 3,5-dimethyl-1H-pyrrole-2,4-dicarboxylate (1)

Acetoacetic ester (40 ml, 320 mmol) and glacial acetic acid (80 ml) were mixed in a flask. The mixture was cooled down to 0 °C with an ice-water bath, and then aqueous solution (16 ml H_2O) of sodium nitrite (10.9 g, 160 mmol) was added dropwise under stirring in 30 min, while keeping the temperature below 10 °C. Reaction mixture was stirred for 2 hours at room temperature before adding zinc powder (20,5 g, 320 mmol). Zinc powder was added portionwise to keep the temperature below 25 °C. After heating to 95 °C and zinc dissolving the reaction mixture was heated to reflux for 1 h. Hot mixture was poured into the ice-water and then beige precipitate was collected by filtration and washed with ice-water and ice-ethanol. The resulting solid was dried under vacuum to provide the product with yield 52%.

^{1}H NMR spectrum (400 MHz, DMSO, δ, ppm): 1.26 (m, 6H), 2.42 (m, 6H), 4.19 (m, 4H), 11.82 (br.s, 1H).

^{13}C NMR spectrum (100 MHz, DMSO, δ, ppm): 11.76; 13.51; 14.29; 14.40; 58.90; 59.50; 112.26; 117.53; 129.52; 139.41; 160.70; 164.61.

Synthesis of 2,4-dimethyl-1H-pyrrole (2)

Diethyl 3,5-dimethyl-1H-pyrrole-2,4-dicarboxylate (14,7 g, 60 mmol) and KOH (19,9 g, 500 mmol) were charged and heated to reflux in ethylene glycol (70 ml) purged with argon for 1,5 h at 160 °C. The mixture was cooled down to room temperature, extracted with CH_2Cl_2 (3 x 150 ml) and then dried over Na_2SO_4. The product was purified by vacuum distillation. A clear colorless liquid of 2,4-dimethylpyrrole was obtained with yield 55%.

^{1}H NMR spectrum (400 MHz, $CDCl_3$, δ, ppm): 2.12 (s, 3H), 2.26 (s, 3H), 5.78 (s, 1H), 6.43 (s, 1H), 7.62 (br. s, 1H).

^{13}C NMR spectrum (100 MHz, $CDCl_3$, δ, ppm): 11.48; 12.60; 107.22; 113.41; 118.71; 127.33.

Synthesis of 4-(5,5-difluoro-1,3,7,9-tetramethyl-5H-4λ^4,5λ^4-dipyrrolo[1,2-c:2',1'-f][1,3,2]diazaborinin-10-yl)phenol (3)

2,4-dimethylpyrrole (0,925 ml, 9,01 mmol) was dissolved in 75 ml distilled THF and stirred for 15 minutes under argon atmosphere. Then p-hydroxybenzaldehyde (0,5 g, 4.505 mmol) was added and stirred for 30 minutes under argon atmosphere. One drop of TFA was added and the solution stirred at r.t. for another 16 hours. Next, solution of 2,3-dichloro-5,6-dicyano-1,4-benzoquinone (1,025 g, 4,505 mmol) in 50 mL distilled THF was added dropwise, and the stirring was continued for 5 hours. Then, the mixture was cooled down to 0 °C with an ice-water bath and 16 mL of DIPEA and 15,5 mL of BF_3*Et_2O were added. After overnight stirring the reaction mixture was washed with water, dried over Na_2SO_4 and evaporated to dryness under vacuum. Additional purification was carried out by flash chromatography with CH_2Cl_2. Resulting solids were dried under vacuum to provide the barn red solids with yield 39 %.

^{1}H NMR spectrum (400 MHz, $CDCl_3$, δ, ppm): 1.45 (s, 6H); 2.56 (s, 6H); 4.97 (br. s, 1H); 5.98 (s, 2H); 6.96 (d, 2H); 7.14 (d, 2H).

^{13}C NMR spectrum (100 MHz, DMSO-d6, δ, ppm): 14.56; 31.06; 55.28; 116.47; 121.53; 124.68; 129.35; 131.66; 143.16; 154.84; 158.60; 206.75.

Synthesis of 5,5-difluoro-1,3,7,9,10-pentamethyl-5H-4λ^4,5λ^4-dipyrrolo[1,2-c:2',1'-f][1,3,2]diazaborinin (S1)

AcCl (764 mg, 9.7 mmol, 1 eqv) was dissolved in 20 ml DCM. The resulting solution was cooled with an ice-bath and then the solution of 2,4-dymethylpyrrole (1.85 g, 19.4 mmol, 2.05 eqv) in 30 ml DCM was added dropwise. The reaction mixture was left at room temperature for 30 minutes. Next, the solution was cooled with an ice-bath again and Et_3N (2.94 g, 29.2 mmol, 3 eqv) was slowly added. The reaction mixture was stirred for 10 minutes under cooling and then BF_3*Et_2O (6.2 g, 43.7 mmol, 4.5 eqv) was added. The resulting solution was left overnight at room temperature. Next, the solution was diluted with 50 ml DCM and washed with saturated $NaHCO_3$ solution (2 x 80 ml) and water (1 x 80 ml). The organic layer was dried over anhydrous Na_2SO_4. The purification of the product S1 was conducted using column chromatography (DCM : petroleum ether = 1:1) to provide the desired product with yield of 53%.

^{1}H NMR spectrum (400 MHz, $CDCl_3$, δ, ppm): 2.42 (s, 6H); 2.53 (s, 6H); 2.59 (s,3H); 6.06 (s, 2H).

Synthesis of 5,5-difluoro-2-formyl-1,3,7,9,10-pentamethyl-5H-5λ⁴,6λ⁴-dipyrrolo[1,2-c:2',1'-f][1,3,2]diazaborinin (S2)

DMF (5 ml) and $POCl_3$ (3 ml) were charged into a flask under cooling in an ice-water bath and stirred under argon atmosphere for 1 hour. BODIPY **S1** (368 mg, 0.88 mmol) was dissolved in 5 mL of DCM and added to the mixture, left to stir overnight. The reaction mixture was slowly poured into a saturated aqueous solution of Na_2CO_3 (200 mL) cooled in an ice-water bath. When pH > 9 was reached, it was left under stirring for one hour, followed by extraction with DCM. The solution was dried over anhydrous Na_2SO_4, then the solvent was removed under reduced pressure. Scarlet-orange powder was obtained and purified by chromatography on silica gel column with eluting solvent of DCM with yield 86%.

^{1}H NMR spectrum (400 MHz, $CDCl_3$, δ, ppm): 2.48 (s, 3H); 2.58 (s, 3H); 2.68 (s, 3H); 2.73 (s, 3H); 2.78 (s, 3H); 6.24 (s, 1H); 10.10 (s, 1H).

Synthesis of 2-(benzo[d]thiazol-2-yl)-5,5-difluoro-1,3,7,9,10-pentamethyl-5H-5λ⁴,6λ⁴-dipyrrolo[1,2-c:2',1'-f][1,3,2]diazaborinin (5-MB-SZ)

2-aminothiophenol (31 mg, 0.25 mmol) and BODIPY **S2** (60 mg, 0.21 mmol) were mixed in DMF (3 mL), then the iodine crystal was added (27 mg, 0.11 mmol). The mixture was heated to 100 °C and stirred for 2 hours. After that the mixture was cooled to room temperature followed by a dilution with DCM and extraction with the 10% solution of sodium thiosulfate. The organic layer was dried over anhydrous Na_2SO_4, then the solvent was removed under reduced pressure. Red-orange crystal product was obtained and purified by chromatography on silica gel column with eluting solvent of DCM with yield 18%.

^{1}H NMR spectrum (400 MHz, $CDCl_3$, δ, ppm): 2.47 (s, 3H); 2.58 (s, 3H); 2.66 (s, 3H); 2.70 (s, 3H); 2.78 (s, 3H); 6.18 (s, 1H); 7.41 (t, 1H); 7.54 (t, 1H); 7.93 (d, 1H); 8.11 (d, 1H).

2. NMR Spectra and HR-/LC-MS data

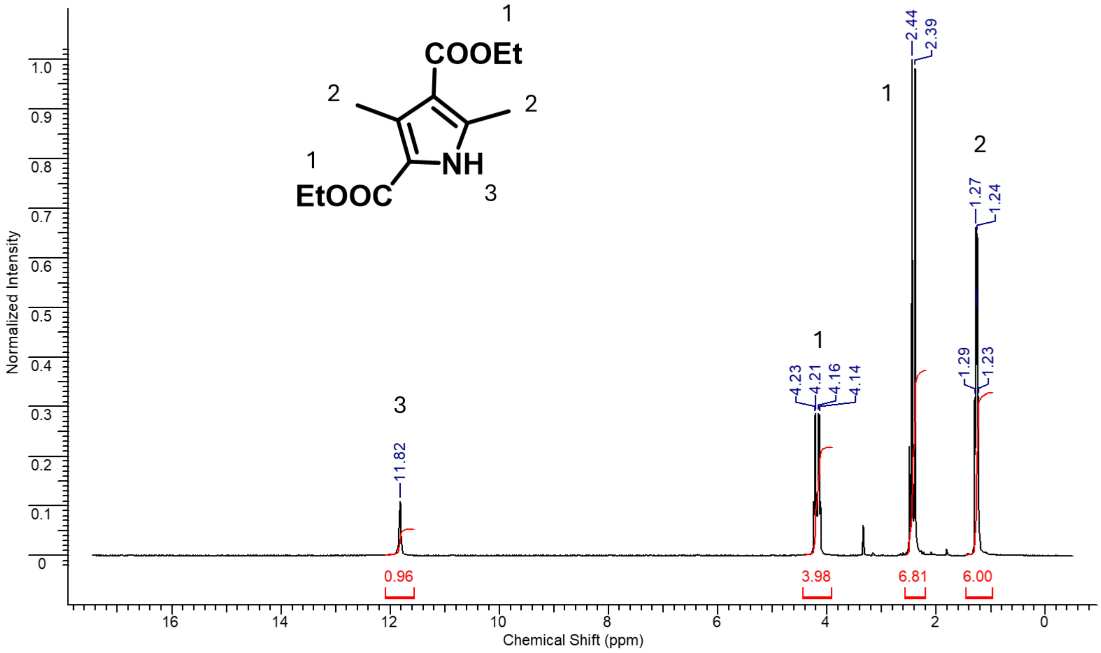

Figure S1. NMR ^{1}H spectrum of diethyl 3,5-dimethyl-1H-pyrrole-2,4-dicarboxylate (**1**) in DMSO-d6

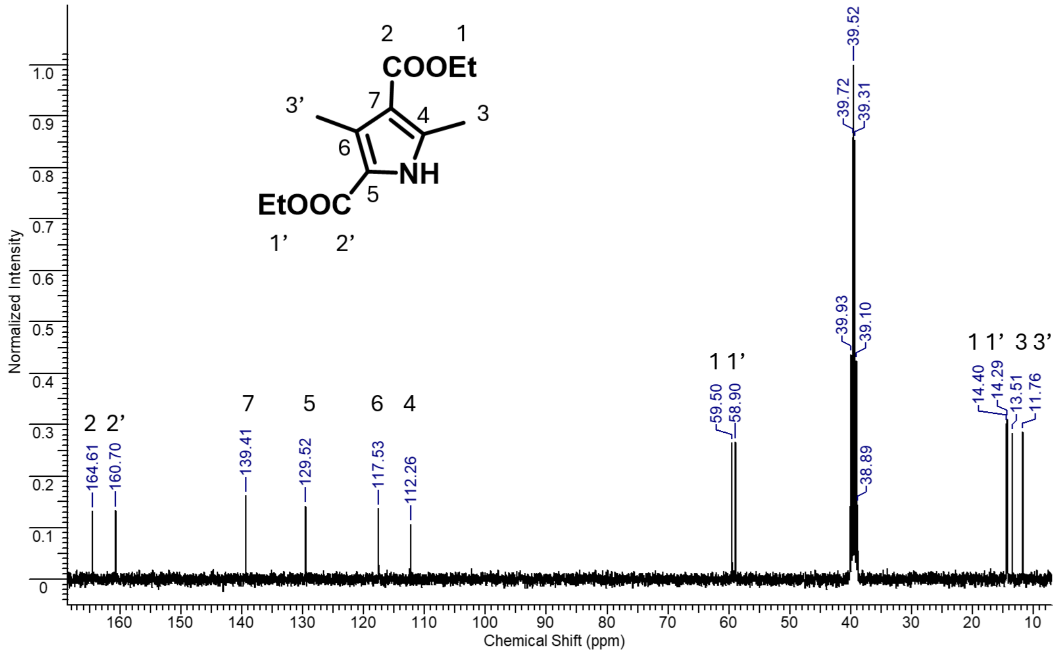

Figure S2. NMR ^{13}C spectrum of diethyl 3,5-dimethyl-1H-pyrrole-2,4-dicarboxylate (**1**) in DMSO-d6

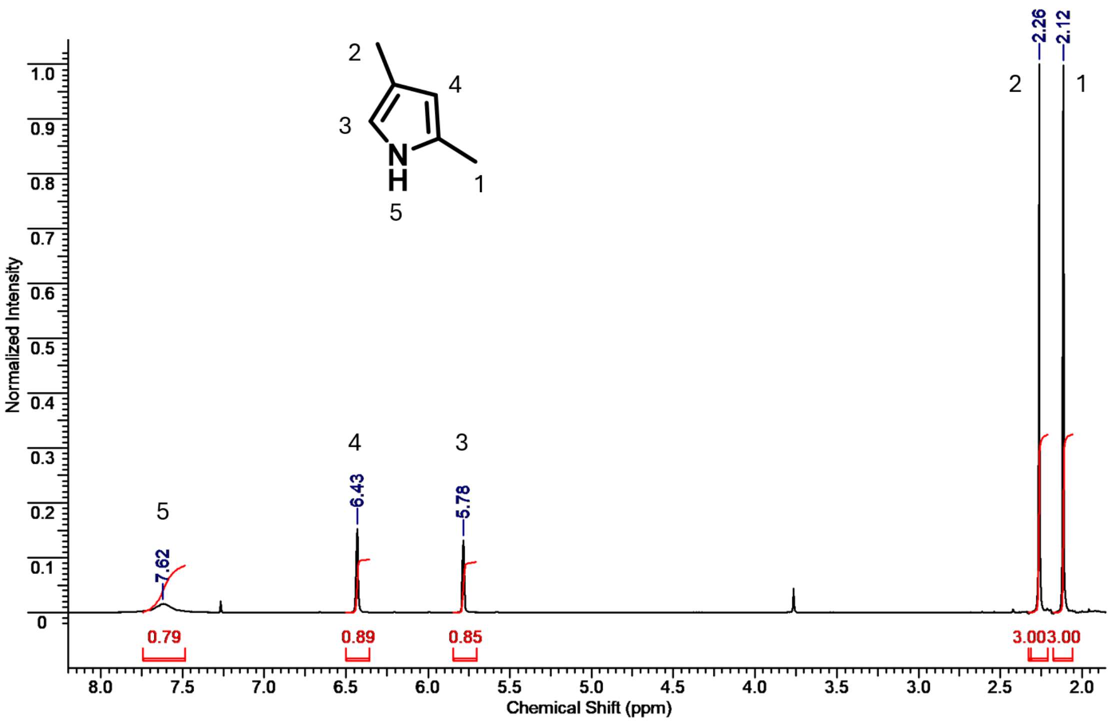

Figure S3. NMR ^{1}H spectrum of 2,4-dimethyl-1H-pyrrole (**2**) in $CDCl_3$

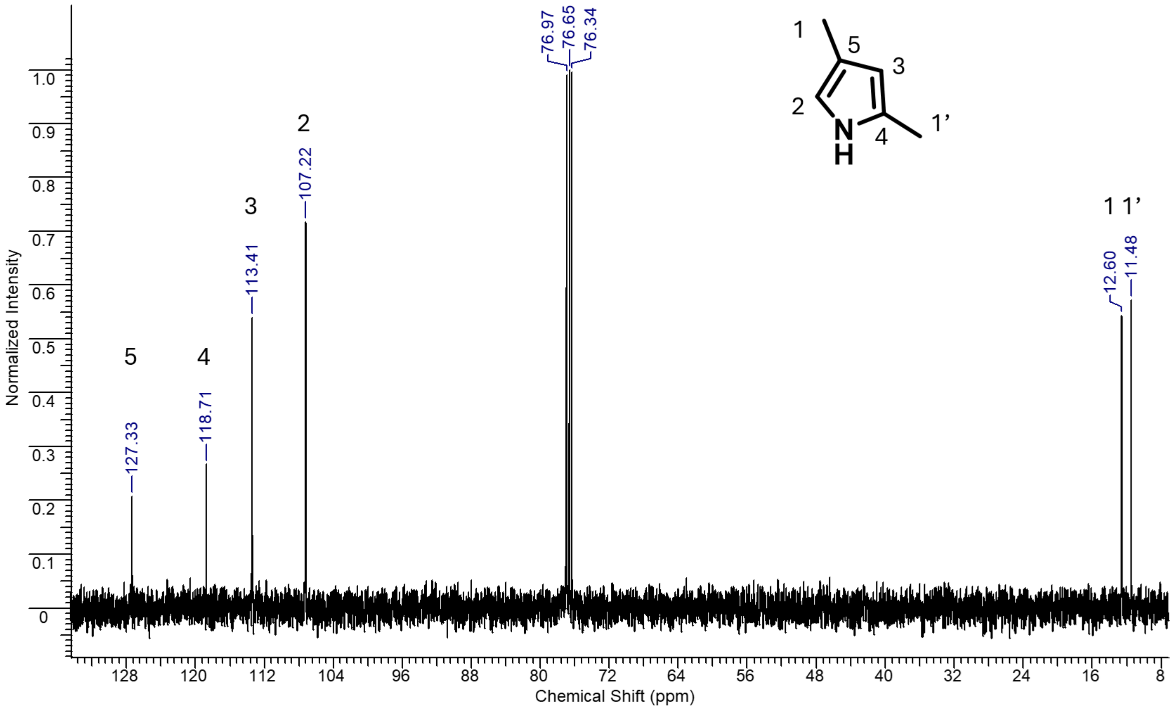

Figure S4. NMR ^{13}C spectrum of 2,4-dimethyl-1H-pyrrole (**2**) in $CDCl_3$

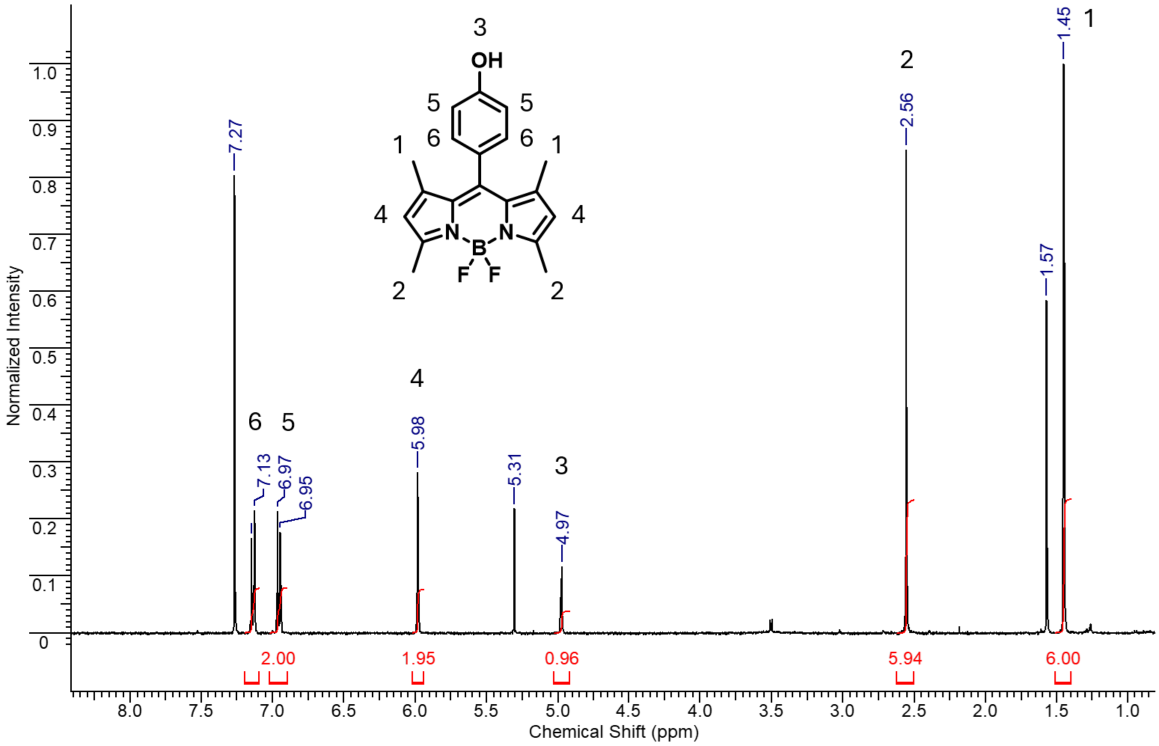

Figure S5. NMR ^{1}H spectrum of 4-(5,5-difluoro-1,3,7,9-tetramethyl-5H-4λ^{4},5λ^{4}-dipyrrolo[1,2-c:2',1'-f][1,3,2]diazaborinin-10-yl)phenol (**3**) in $CDCl_3$

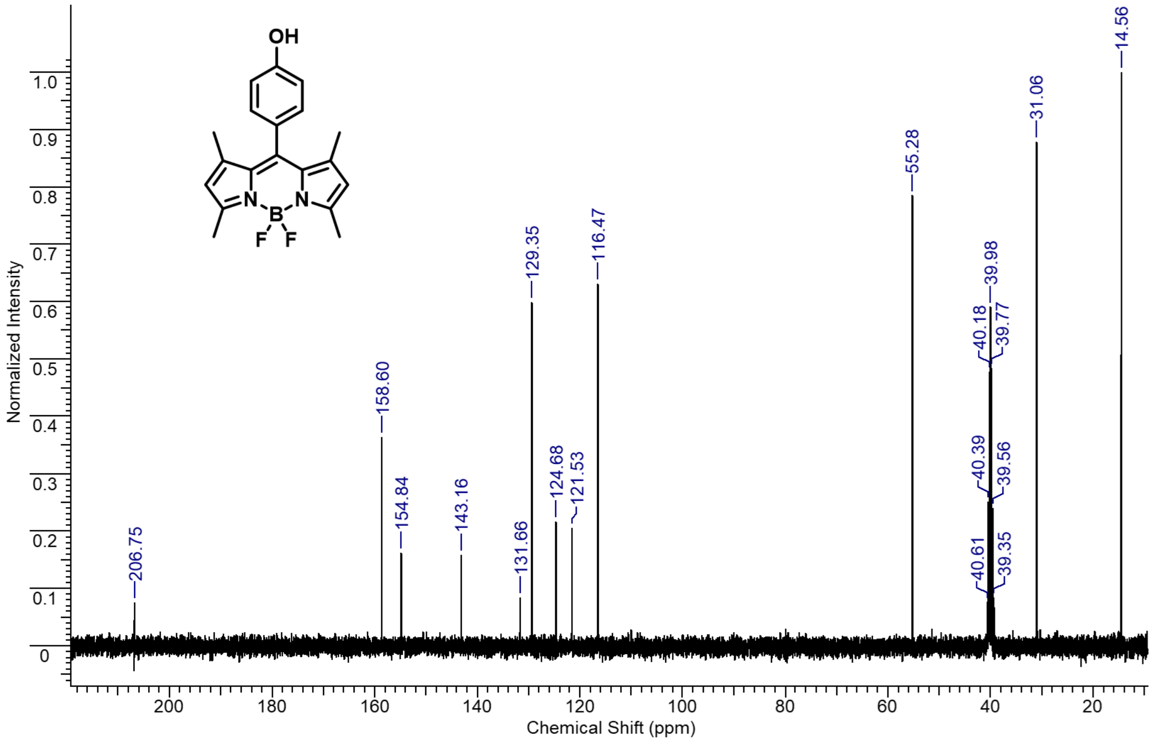

Figure S6. NMR ^{13}C spectrum of 4-(5,5-difluoro-1,3,7,9-tetramethyl-5H-4λ^{4},5λ^{4}-dipyrrolo [1,2-c:2',1'-f][1,3,2]diazaborinin-10-yl)phenol (**3**) in DMSO-d6

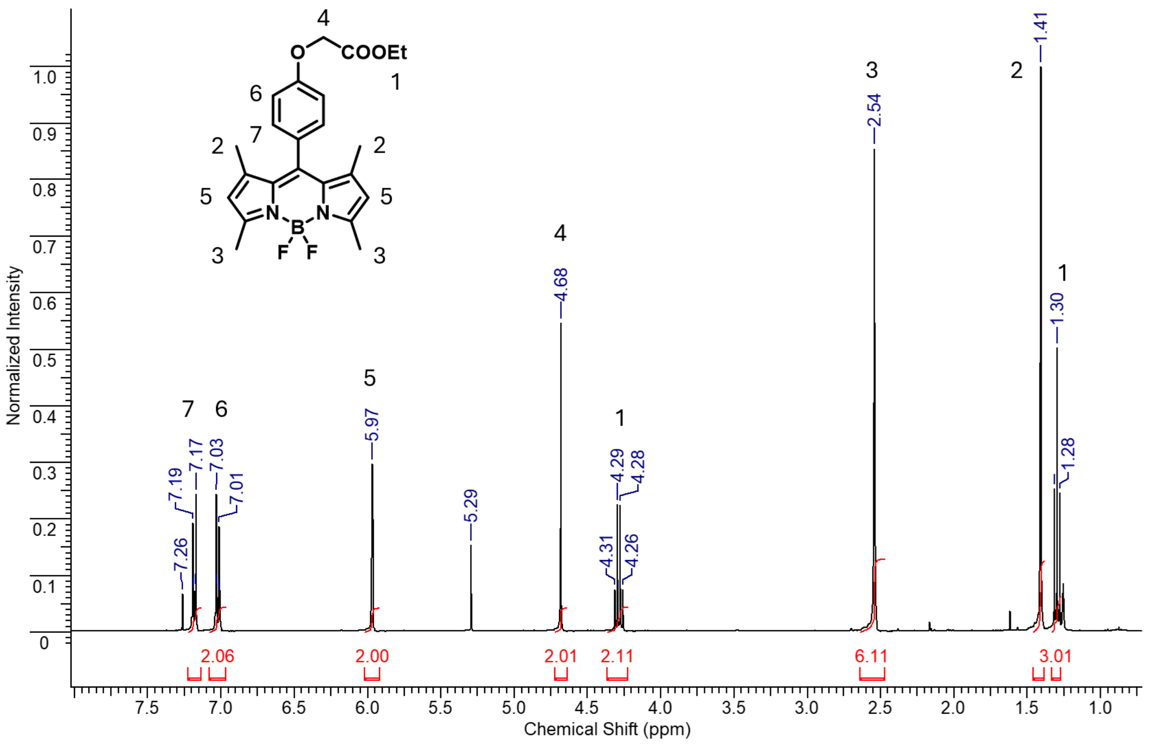

Figure S7. NMR ^{1}H spectrum of ethyl 2-(4-(5,5-difluoro-1,3,7,9-tetramethyl-5H-4λ^{4},5λ^{4}-dipyrrolo[1,2-c:2',1'-f][1,3,2]diazaborinin-10-yl)phenoxy)acetate (**4**) in $CDCl_3$

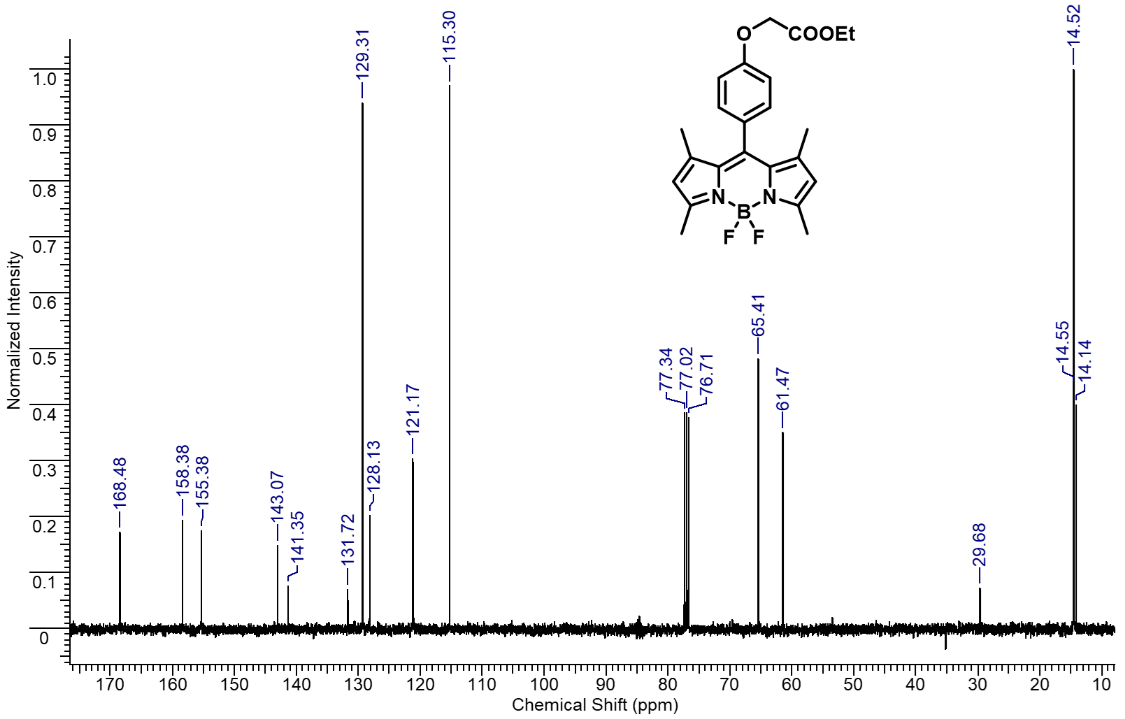

Figure S8. NMR ^{13}C spectrum of ethyl 2-(4-(5,5-difluoro-1,3,7,9-tetramethyl-5H-4λ^{4},5λ^{4}-dipyrrolo[1,2-c:2',1'-f][1,3,2]diazaborinin-10-yl)phenoxy)acetate (**4**) in $CDCl_3$

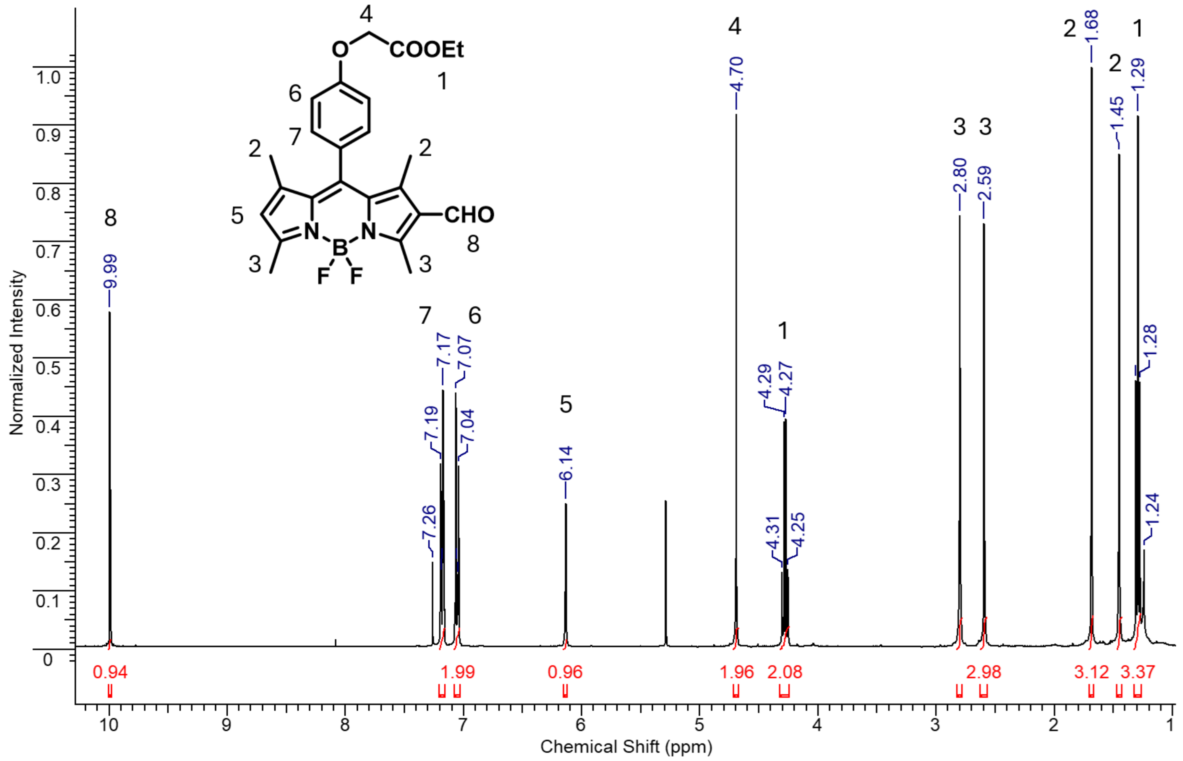

Figure S9. NMR ^{1}H spectrum of ethyl 2-(4-(5,5-difluoro-2-formyl-1,3,7,9-tetramethyl-5H-5λ^{4},6λ^{4}-dipyrrolo[1,2-c:2',1'-f][1,3,2]diazaborinin-10-yl)phenoxy)acetate (**5**) in $CDCl_3$

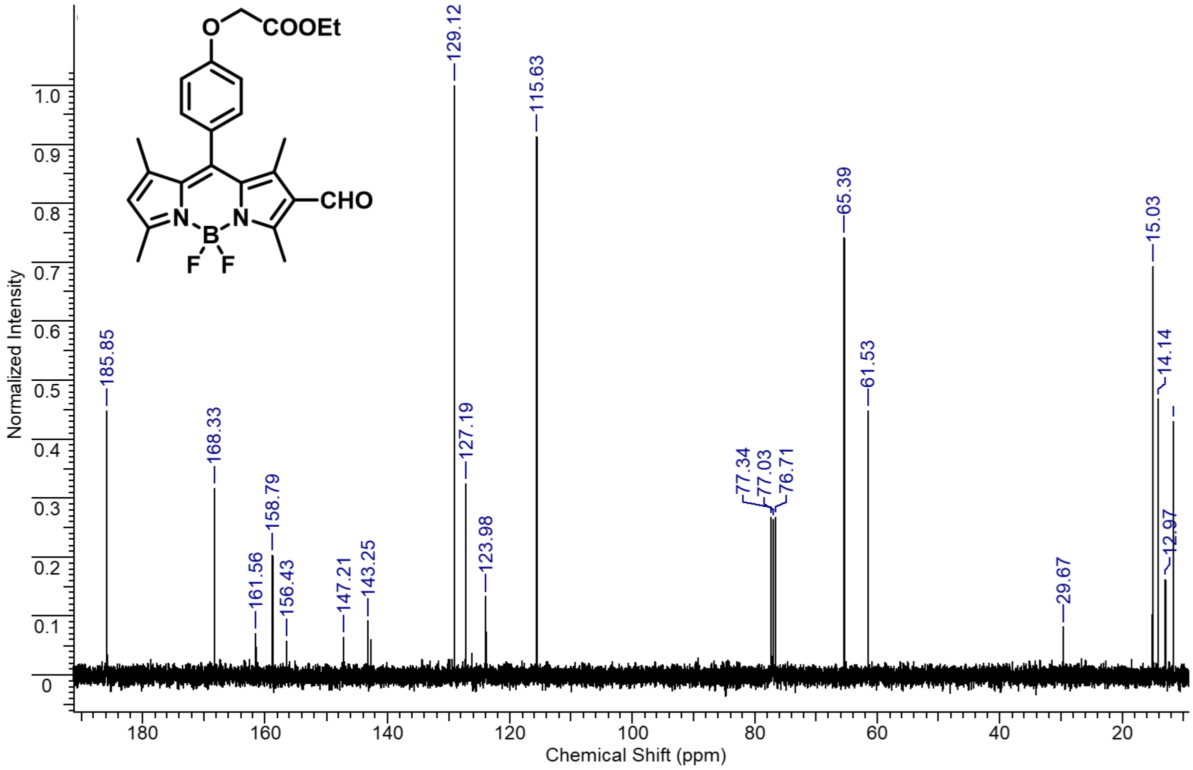

Figure S10. NMR ^{13}C spectrum of ethyl 2-(4-(5,5-difluoro-2-formyl-1,3,7,9-tetramethyl-5H-5λ^{4},6λ^{4}-dipyrrolo[1,2-c:2',1'-f][1,3,2]diazaborinin-10-yl)phenoxy) acetate (**5**) in $CDCl_3$

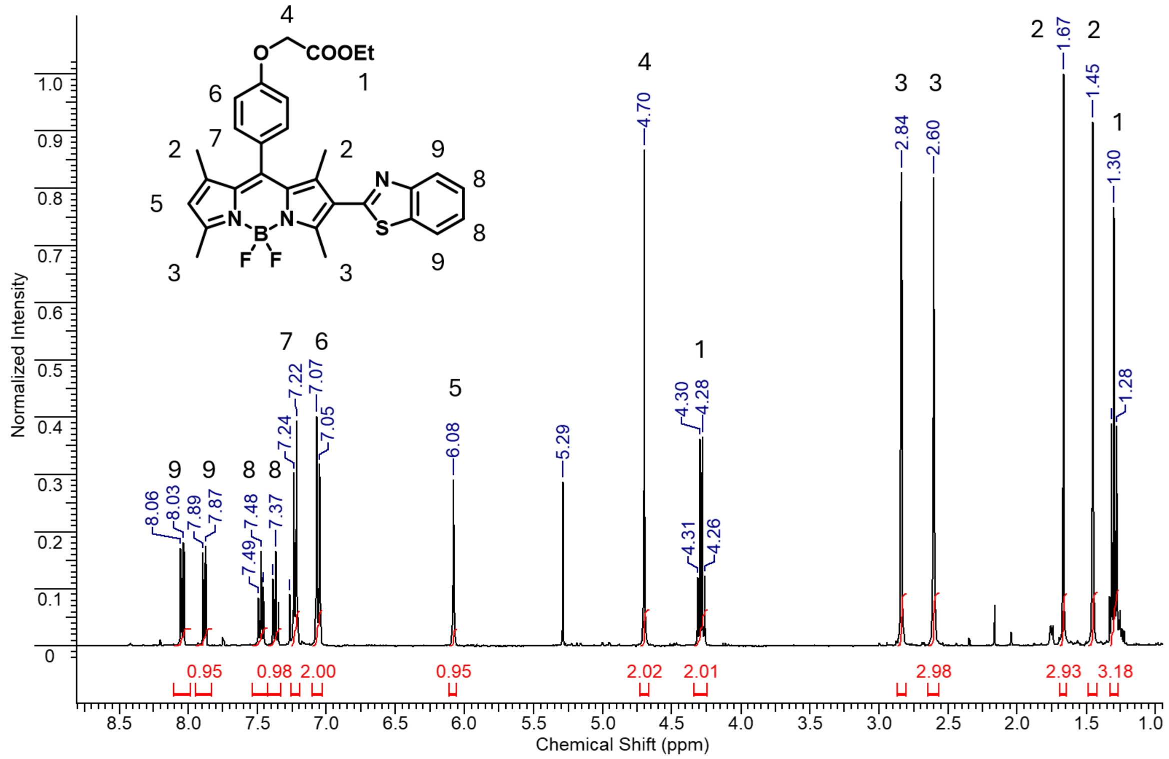

Figure S11. NMR ^{1}H spectrum of ethyl 2-(4-(2-(benzo[d]thiazol-2-yl)-5,5-difluoro-1,3,7,9-tetramethyl-5H-5λ^{4},6λ^{4}-dipyrrolo[1,2-c:2',1'-f][1,3,2]diazaborinin-10-yl) phenoxy) acetate (**6**) in $CDCl_3$

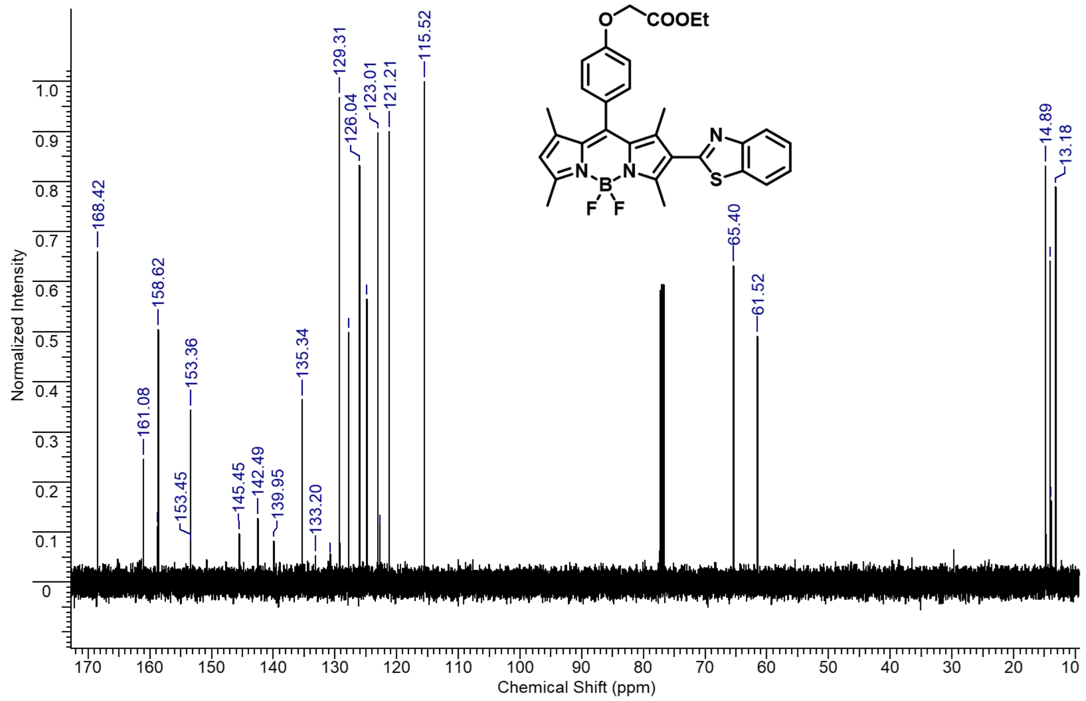

Figure S12. NMR ^{13}C spectrum of ethyl 2-(4-(2-(benzo[d]thiazol-2-yl)-5,5-difluoro-1,3,7,9-tetramethyl-5H-5λ^4,6λ^4-dipyrrolo[1,2-c:2',1'-f][1,3,2]diazaborinin-10-yl) phenoxy)acetate (**6**) in $CDCl_3$

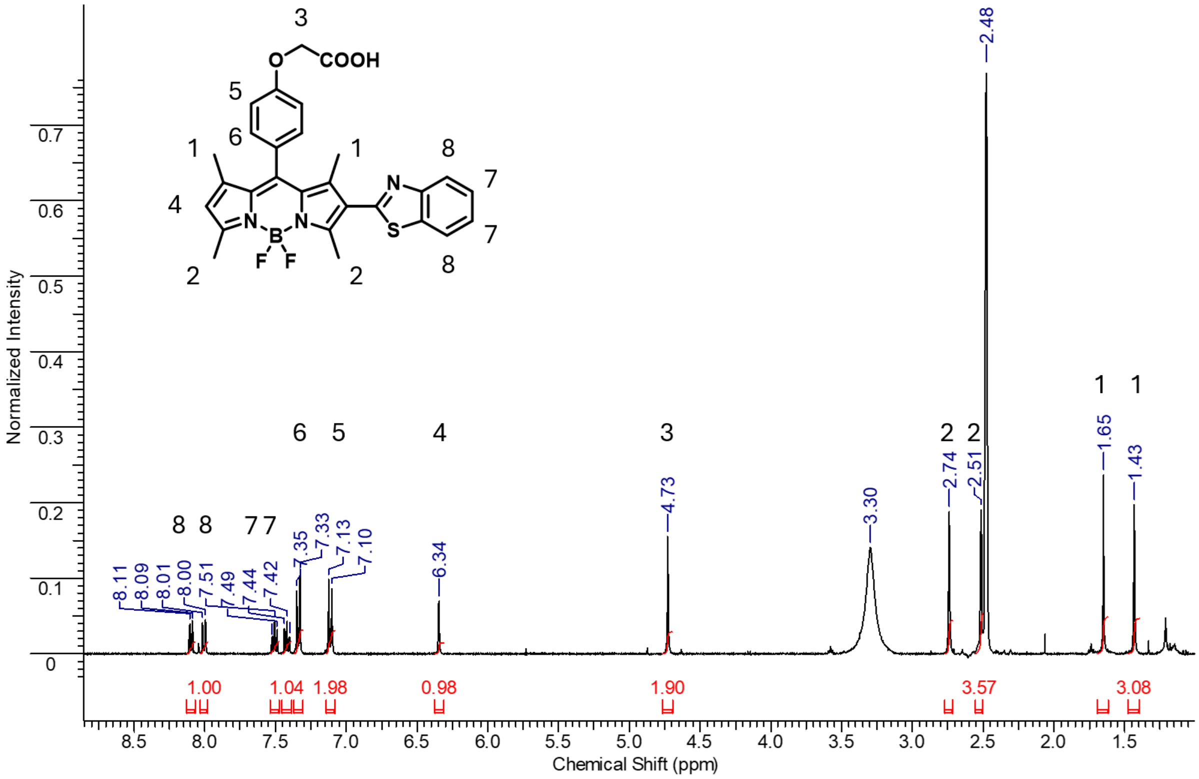

Figure S13. NMR ^1H spectrum of 2-(4-(2-(benzo[d]thiazol-2-yl)-5,5-difluoro-1,3,7,9-tetramethyl-5H-5λ^4,6λ^4-dipyrrolo[1,2-c:2',1'-f][1,3,2]diazaborinin-10-yl)phenoxy)acetic acid (**7**) in DMSO-d6

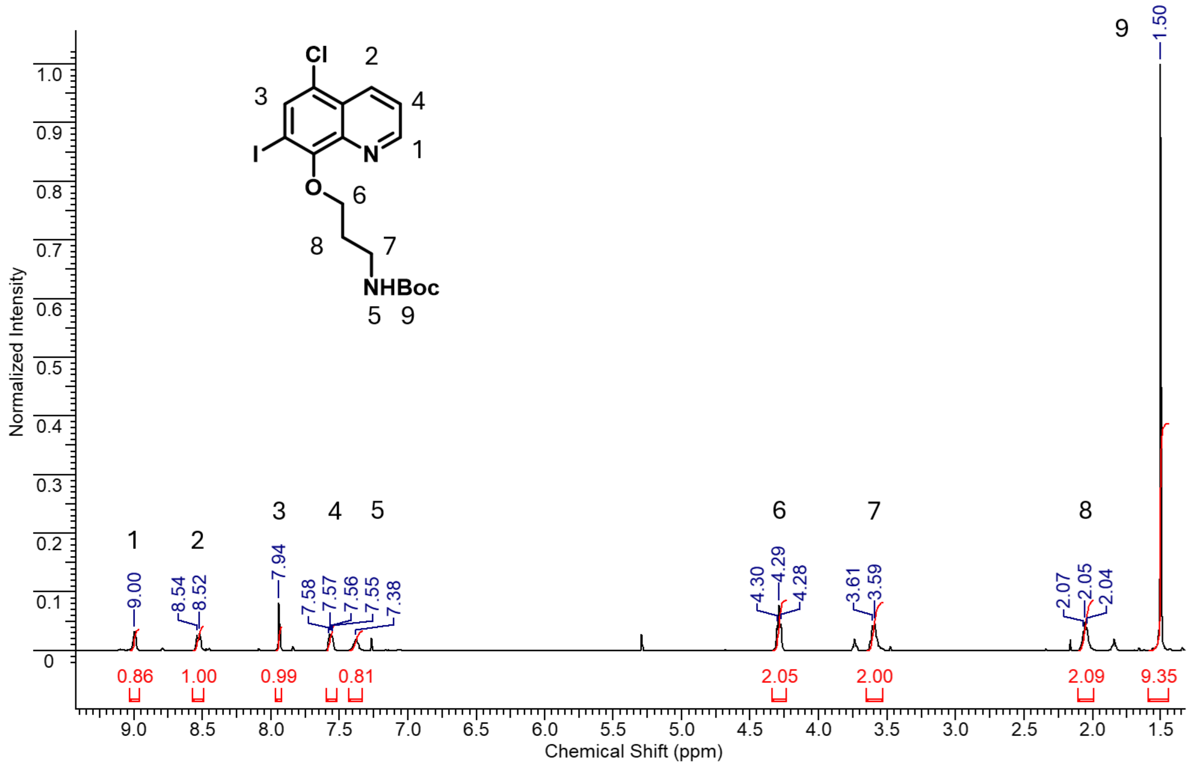

Figure S14. NMR ^{1}H spectrum of tert-butyl (3-((5-chloro-7-iodoquinolin-8-yl)oxy)propyl)carbamate (**8**) in $CDCl_3$

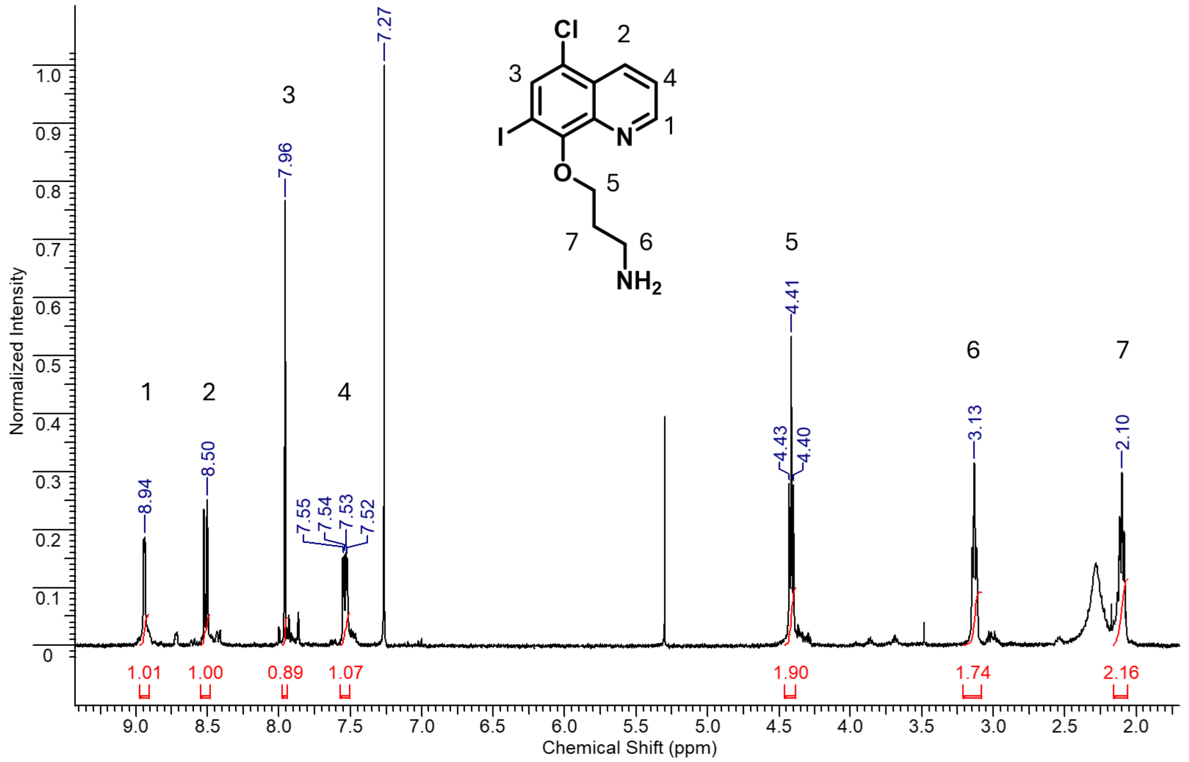

Figure S15. NMR ^{1}H spectrum of tert-butyl 3-((5-chloro-7-iodoquinolin-8-yl)oxy)propan-1-amine (**9**) in $CDCl_3$

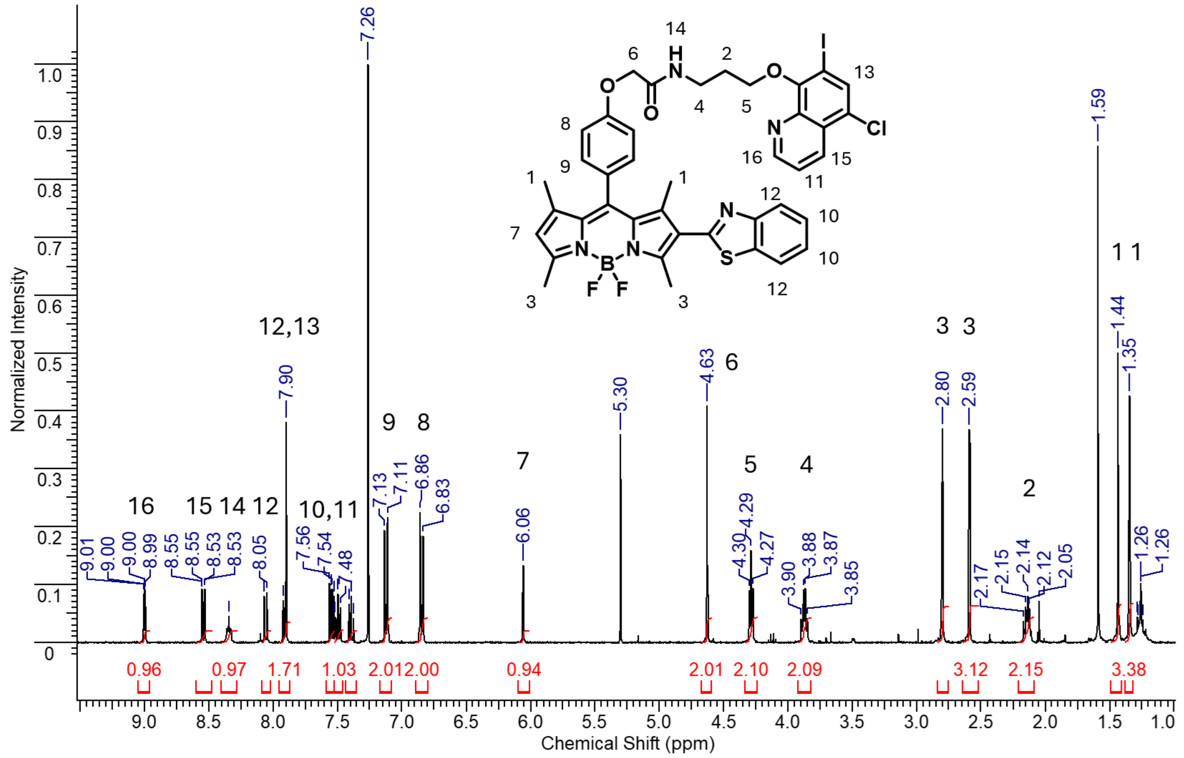

Figure S16. NMR ^{1}H spectrum of N-(3-((5-chloro-7-iodoquinolin-8-yl)oxy)propyl)-2-(4-(5,5-difluoro-1,3,7,9-tetramethyl-5H-4l4,5l4-dipyrrolo[1,2-c:2',1'-f][1,3,2] diazaborinin-10-yl)phenoxy)acetamide (**BDP-CLQ**) in $CDCl_3$

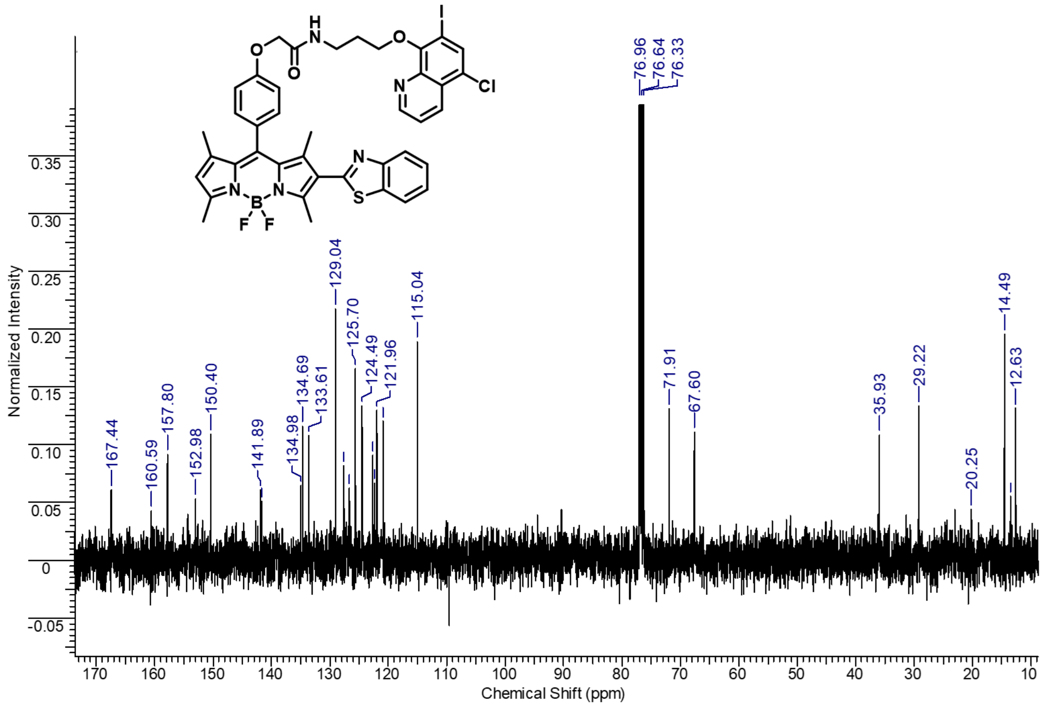

Figure S17. NMR ^{13}C spectrum of N-(3-((5-chloro-7-iodoquinolin-8-yl)oxy)propyl)-2-(4-(5,5-difluoro-1,3,7,9-tetramethyl-5H-4l4,5l4-dipyrrolo[1,2-c:2',1'-f][1,3,2] diazaborinin-10-yl)phenoxy)acetamide (**BDP-CLQ**) in $CDCl_3$

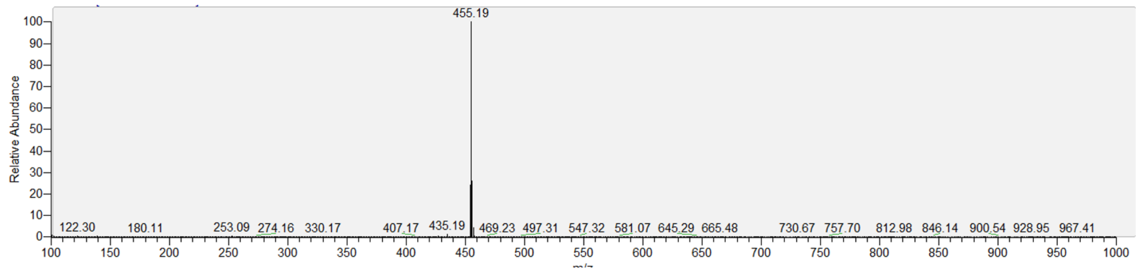

Figure S18. LCMS data on ethyl 2-(4-(5,5-difluoro-2-formyl-1,3,7,9-tetramethyl-5H-5λ^4,6λ^4-dipyrrolo[1,2-c:2',1'-f][1,3,2]diazaborinin-10-yl)phenoxy) acetate (**5**)

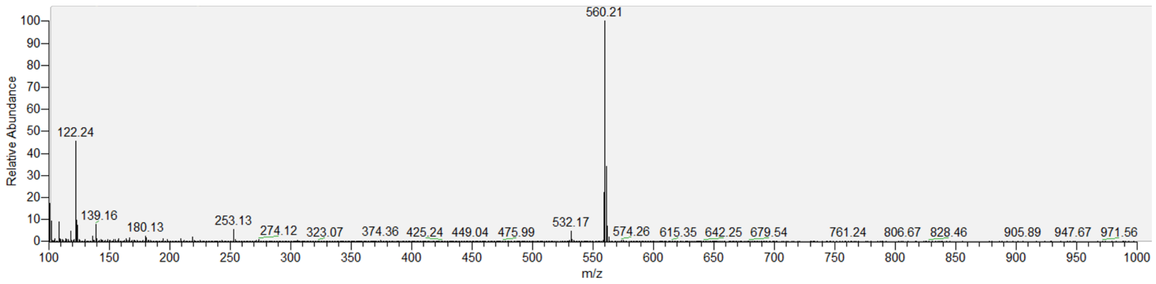

Figure S19. LCMS data on ethyl 2-(4-(2-(benzo[d]thiazol-2-yl)-5,5-difluoro-1,3,7,9-tetramethyl-5H-5λ^4,6λ^4-dipyrrolo[1,2-c:2',1'-f][1,3,2]diazaborinin-10-yl) phenoxy) acetate (**6**)

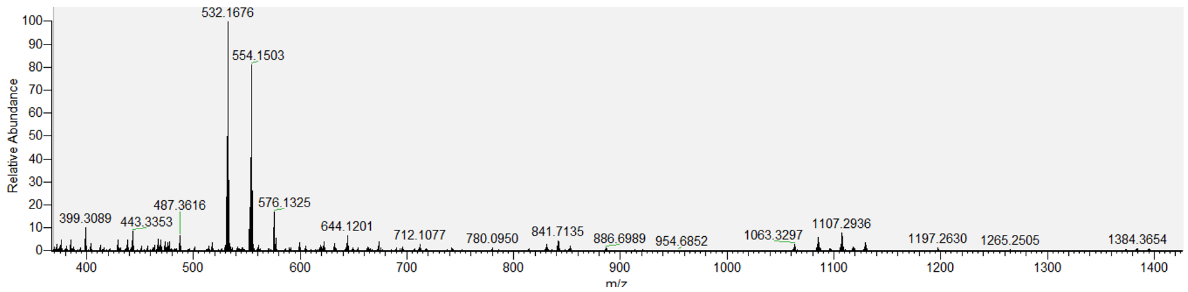

Figure S20. HRMS data on 2-(4-(2-(benzo[d]thiazol-2-yl)-5,5-difluoro-1,3,7,9-tetramethyl-5H-5λ^4,6λ^4-dipyrrolo[1,2-c:2',1'-f][1,3,2]diazaborinin-10-yl)phenoxy)acetic acid (**7**)

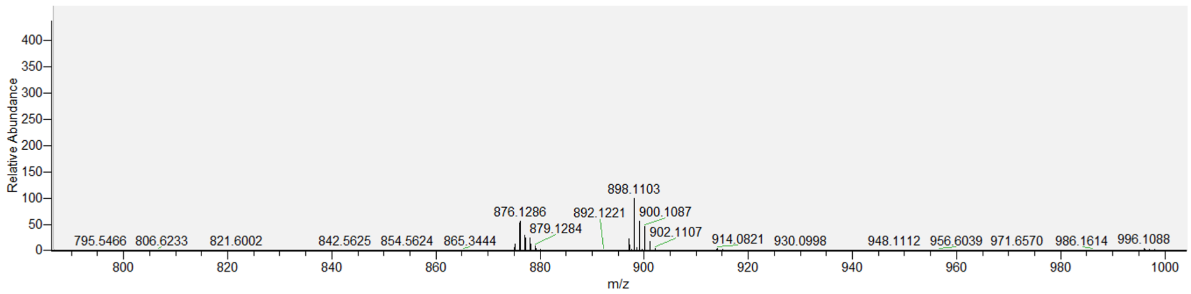

Figure S21. HRMS data on N-(3-((5-chloro-7-iodoquinolin-8-yl)oxy)propyl)-2-(4-(5,5-difluoro-1,3,7,9-tetramethyl-5H-4l4,5l4-dipyrrolo[1,2-c:2',1'-f][1,3,2] diazaborinin-10-yl)phenoxy)acetamide (**BDP-CLQ**)

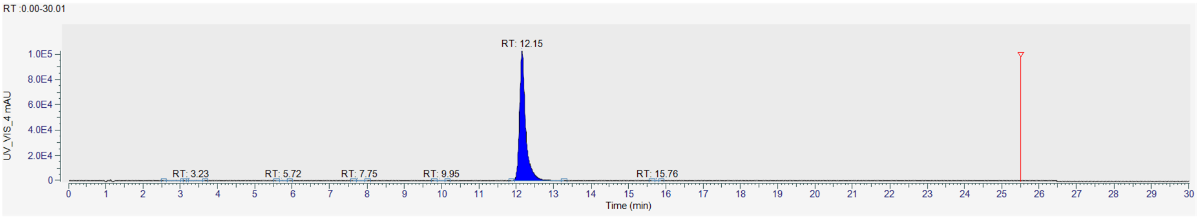

Пик	Время удерживания, мин	Площадь	Площадь, %
1	2,91	1198	0,10%
2	3,23	1580	0,13%
3	5,72	1241	0,10%
4	7,75	497	0,04%
5	9,95	1048	0,09%
6	12,15	1199073	99,47%
7	15,76	878	0,07%

Figure S22. HPLC analysis of N-(3-((5-chloro-7-iodoquinolin-8-yl)oxy)propyl)-2-(4-(5,5-difluoro-1,3,7,9-tetramethyl-5H-4l4,5l4-dipyrrolo[1,2-c:2',1'-f][1,3,2] diazaborinin-10-yl)phenoxy)acetamide (**BDP-CLQ**)

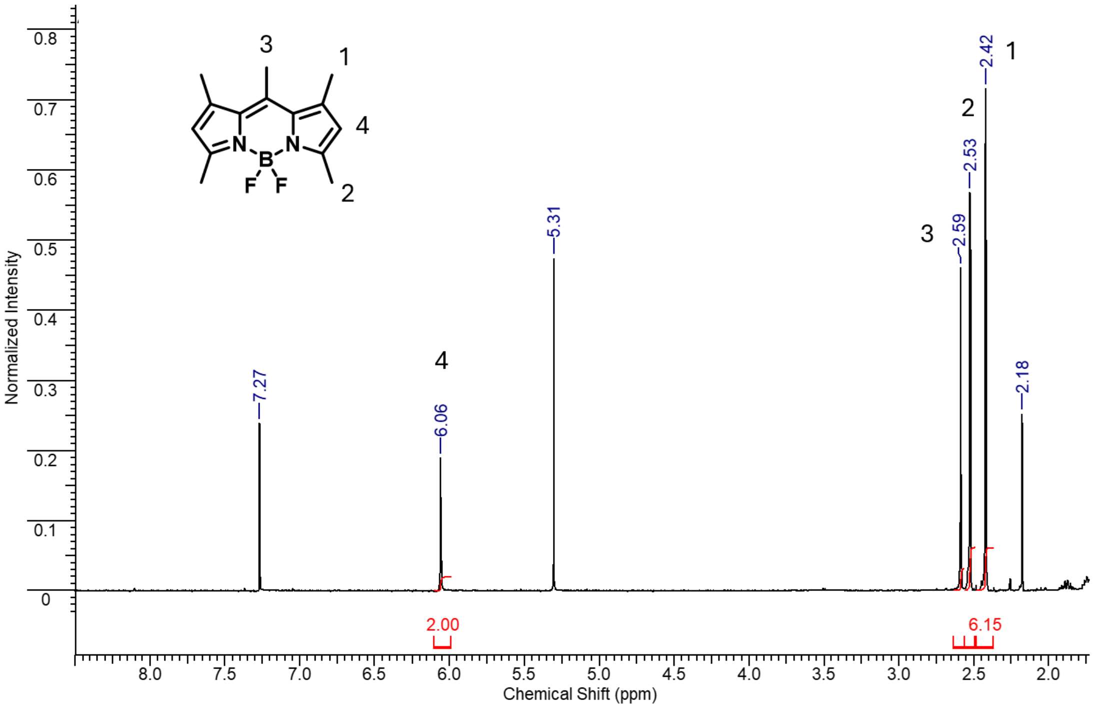

Figure S23. NMR ^{1}H spectrum of 5,5-difluoro-1,3,7,9,10-pentamethyl-5H-4λ^4,5λ^4-dipyrrolo[1,2-c:2',1'-f][1,3,2]diazaborinin (**S1**)

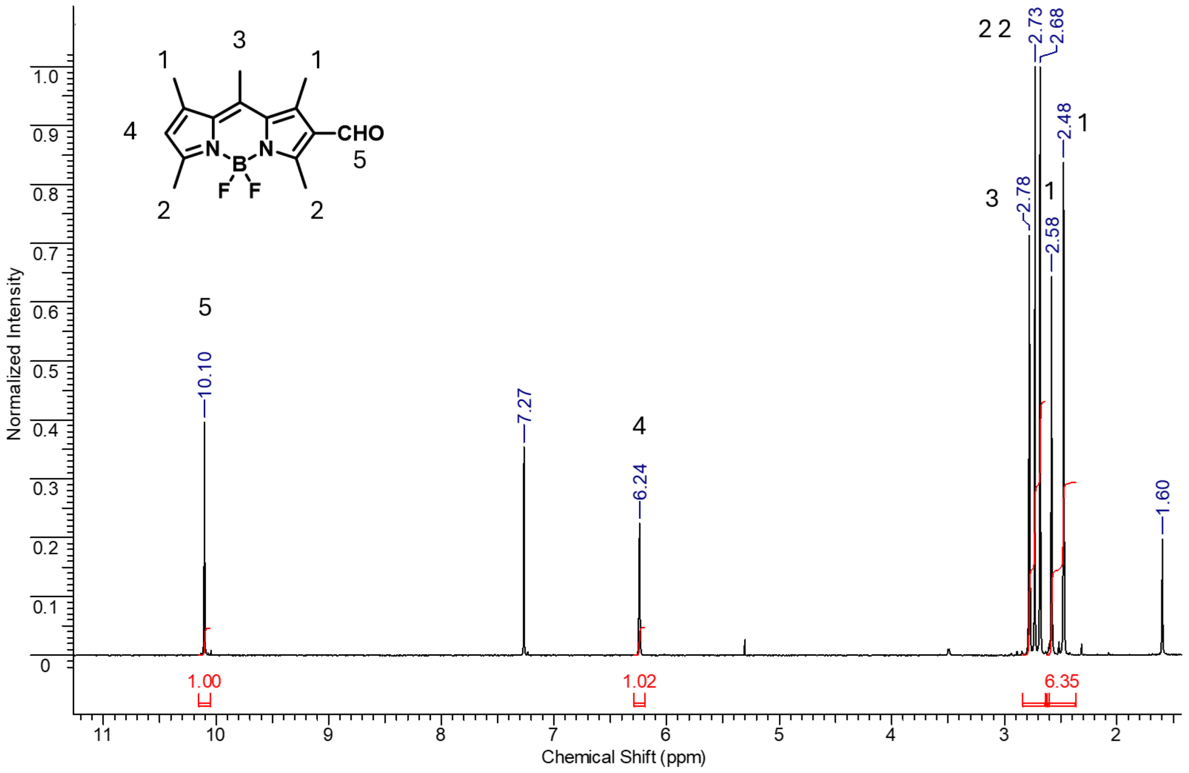

Figure S24. NMR ^{1}H spectrum of 5,5-difluoro-2-formyl-1,3,7,9,10-pentamethyl-5H-5λ^4,6λ^4-dipyrrolo[1,2-c:2',1'-f][1,3,2]diazaborinin (**S2**)

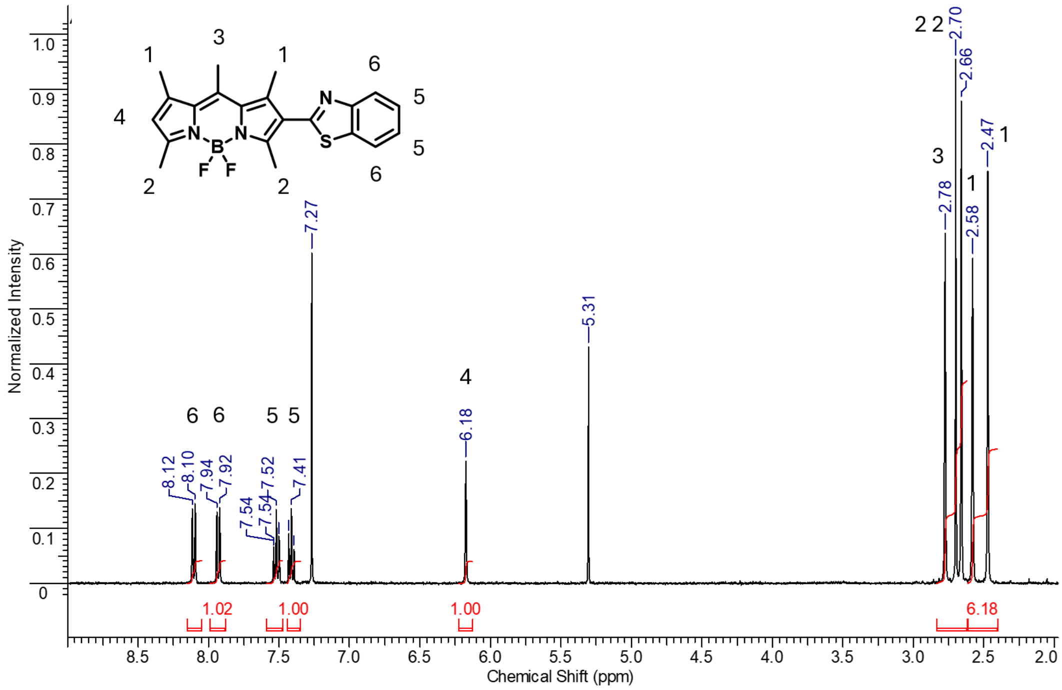

Figure S25. NMR ^{1}H spectrum of 2-(benzo[d]thiazol-2-yl)-5,5-difluoro-1,3,7,9,10-pentamethyl-5H-5λ^4,6λ^4-dipyrrolo[1,2-c:2',1'-f][1,3,2]diazaborinin (**5-MB-SZ**)

3. Copper chelating properties of BDP-CLQ

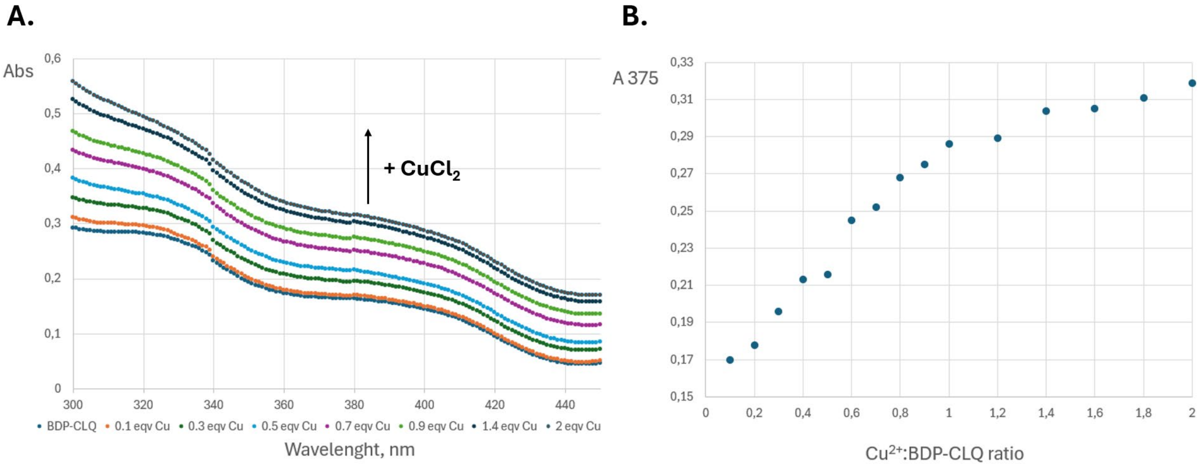

Figure S26. UV-Vis titration of BDP-CLQ with CuCl2 solution. The influence of copper ions concentration increase on the optical density of BDP-CLQ solution (A) and the dependence of absorption of BDP-CLQ solution at 375 nm on Cu^{2+}:BDP-CLQ ratio (B).

4. Cytotoxicity data

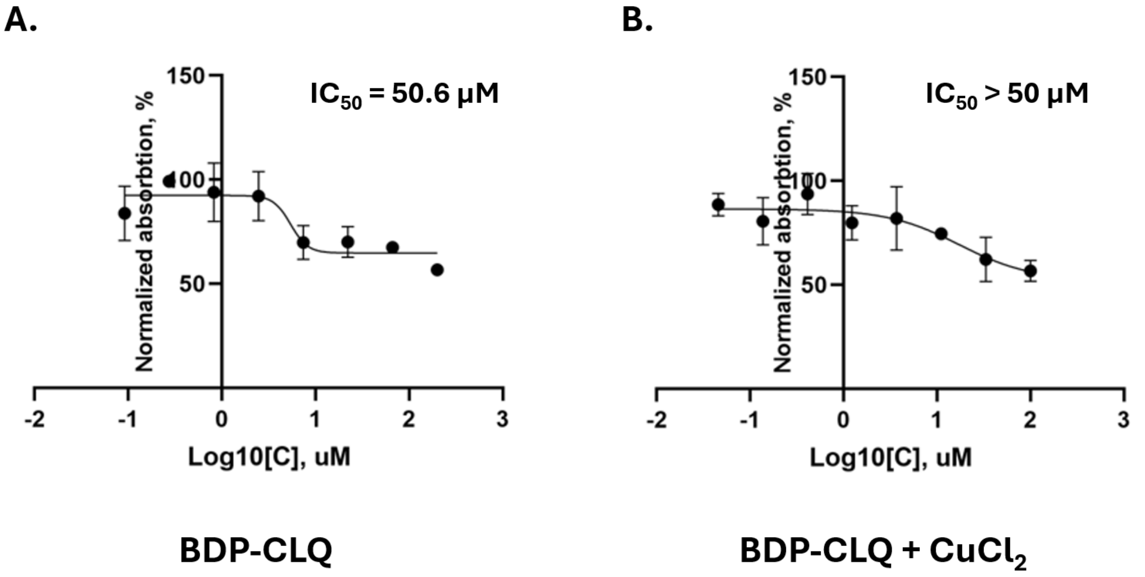

Figure S27. Cytotoxicity data on BDP-CLQ (A) and BDP-CLQ in the presence of equimolar amount of $CuCl_2$ (B) on SH-SY5Y cell line

5. BDP-CLQ titration with Aβ$_{42}$ fibrils solution

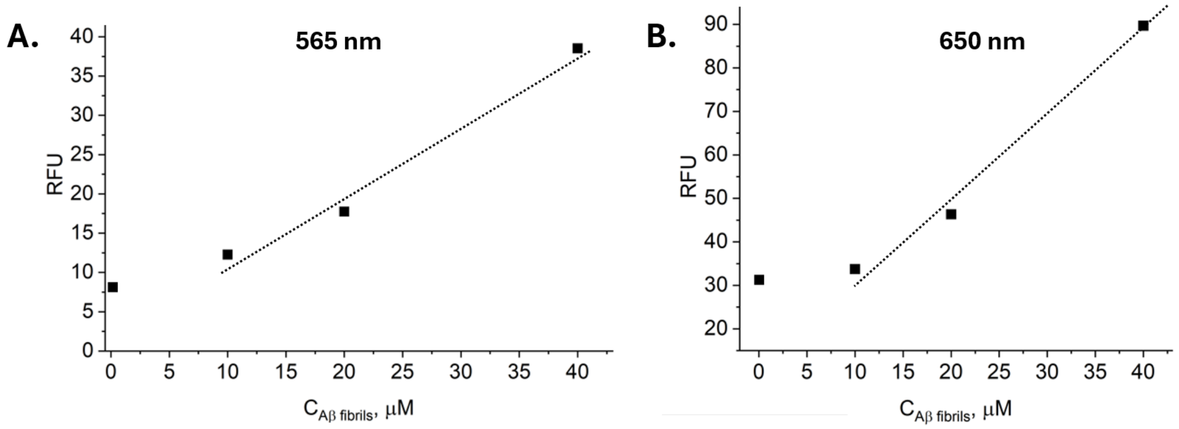

Figure S28. The linear dependence of BDP-CLQ fluorescence enhancement upon titration with Aβ$_{42}$ fibrils at 565 nm (A) and at 650 nm (B)

6. Binding affinity assay of BDP-CLQ to Aβ$_{42}$ fibrils

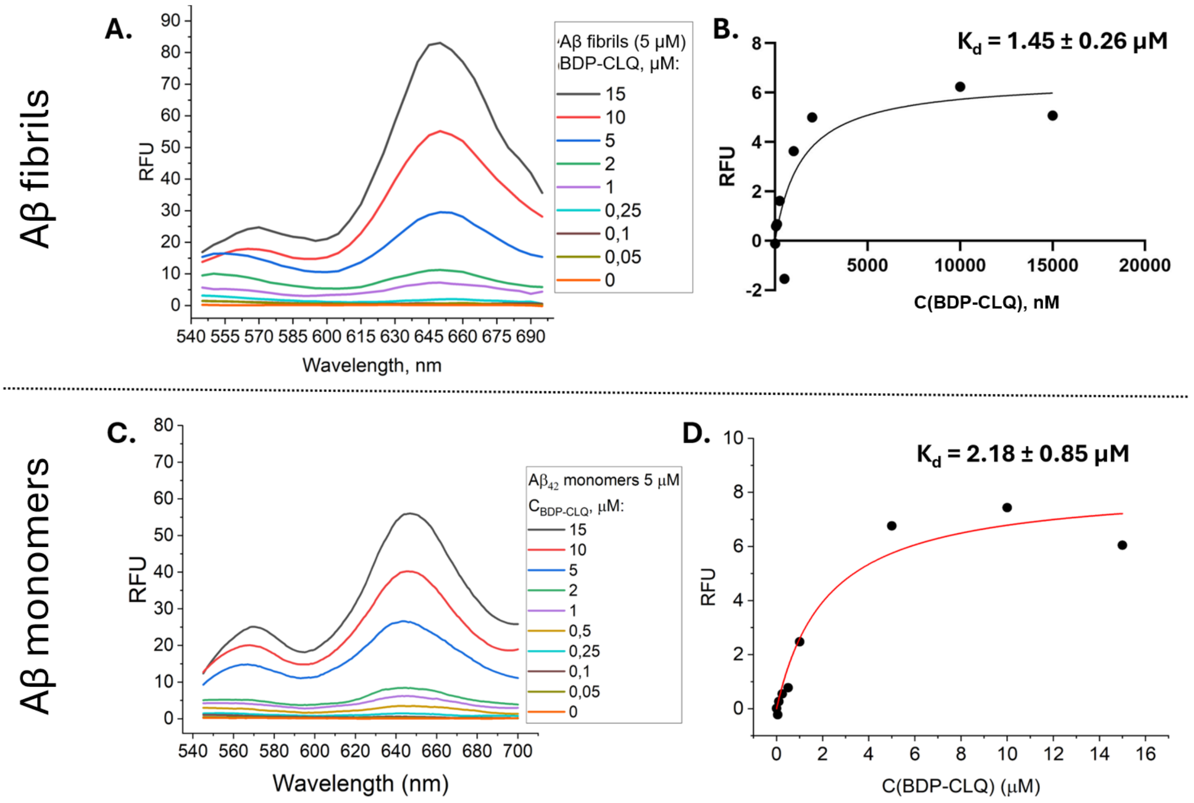

Figure S29. Binding affinity assay of BDP-CLQ to Aβ$_{42}$ fibrils and monomers. Fluorescence enhancement of Aβ$_{42}$ fibrils and monomers solution upon titration with

BDP-CLQ (A and C, respectively) and the dependence of solution fluorescence intensities on BDP-CLQ concentration (B and D)

7. Isothermal calorimetry titration assay

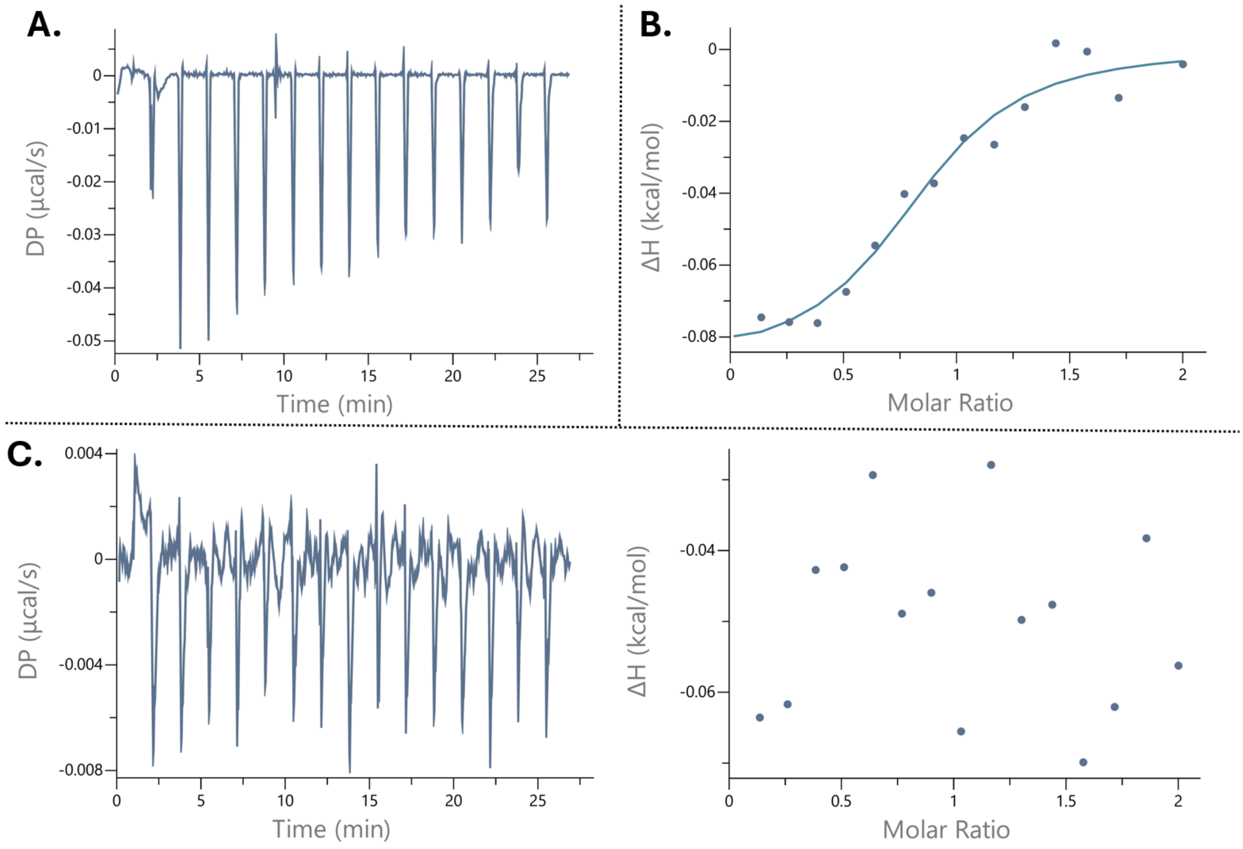

Figure S30. The titration curve of BDP-CLQ – Aβ_{42} monomers isothermal calorimetry titration (A), the dependence of overall BDP-CLQ-Aβ_{42} monomers interaction heat effect on the BDP-CLQ:Aβ_{42} molar ratio (B) and the dilution curve of BDP-CLQ (C)

8. AFM imaging of BDP-CLQ inhibition of $A\beta_{42}$ aggregation process

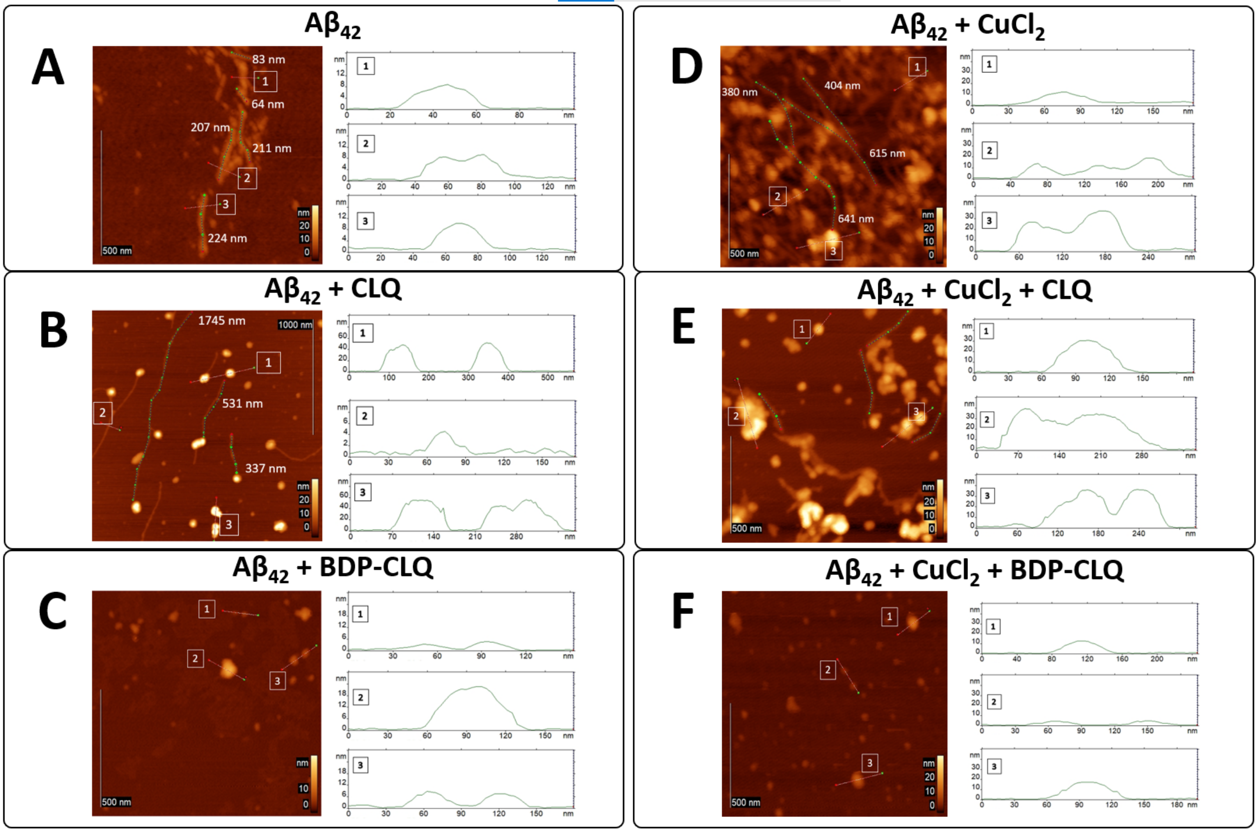

Figure S31. Zoom-in AFM images with profiles and sizes of objects: $A\beta_{42}$ fibrils (25 μM) (A), $A\beta_{42}$ with CLQ (25 μM) (B), $A\beta_{42}$ with BDP-CLQ (50 μM) (C), Cu-induced $A\beta_{42}$ fibrils (25 μM) (D), $A\beta_{42}$ with Cu^{2+} (25 μM) and CLQ (25 μM) (E), and $A\beta_{42}$ with Cu^{2+} (25 μM) and BDP-CLQ (50 μM) (F).

9. *In vivo* visualization of Aβ_{42} species

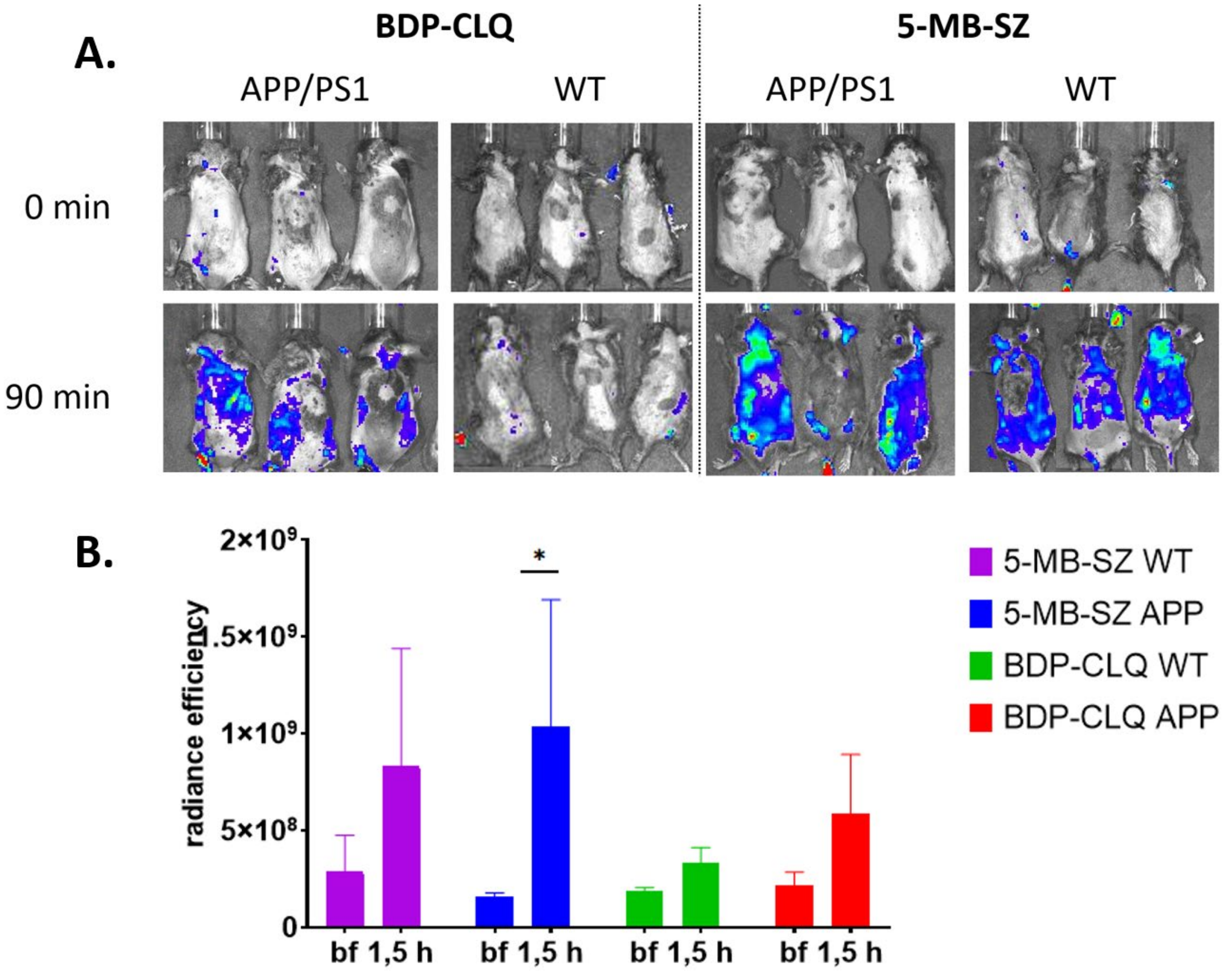

Figure S32. A. IVIS-imaging of fluorescence intensity of APP/PS1 and WT mice administered the 22 μM/kg dose of BDP-CLQ or 5-MB-SZ probe. **B.** Radiant efficiency of head area before (bf) and after injection of BDP-CLQ or 5-MB-SZ probe, * $p < 0.05$ (ANOVA test).

10. BDP-CLQ biodistribution assay

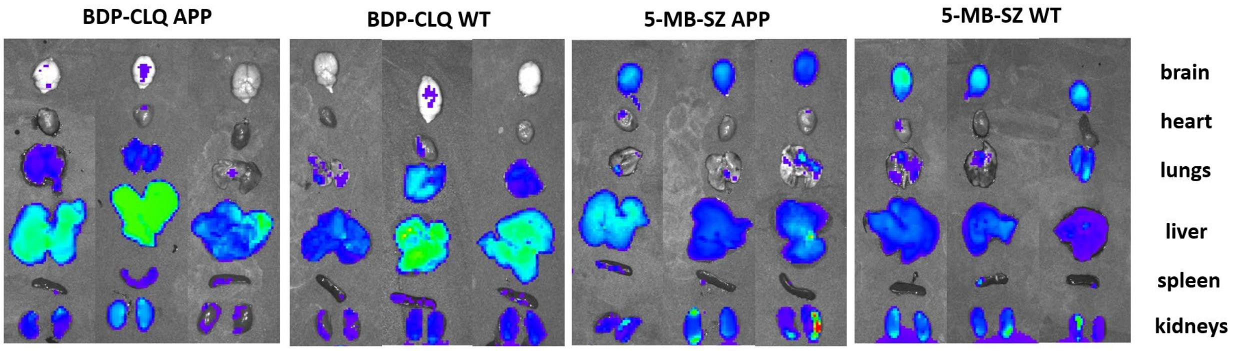

Figure S33. The fluorescence intensities of perfused organs of APP/PS1 and WT mice.
